# Deciphering fibroblast and myeloid-cell states in keloids: an integrative analysis identifies ANKRD1 as a ferroptosis-related candidate gene

**DOI:** 10.3389/fimmu.2026.1872427

**Published:** 2026-10-07

**Authors:** Rongrong Xie, Wanting Yao, Peipei Chen, Xianying Zhou, Xingong Lin, Xinyu Jia, Yuyang Lin, Zehao Yu, Shijie Tang, Caixiang Chen, Penghong Chen, Chaoyang Wang

**Affiliations:** 1 Department of Plastic Surgery, The Second Affiliated Hospital of Fujian Medical University, Quanzhou, China; 2 Department of Obstetrics, The Second Affiliated Hospital of Fujian Medical University, Quanzhou, China; 3 Laboratory for Molecular Cancer Biology, Center for Cancer Biology, VIB, Leuven, Belgium; 4 Department of Breast Surgery, Fujian Medical University Union Hospital, Fuzhou, Fujian, China; 5 Fujian Key Laboratory of Lung Stem Cells, The Second Affiliated Hospital of Fujian Medical University, Quanzhou, Fujian, China; 6 NHC Key Laboratory of Etiological Epidemiology of Chronic Diseases with High Incidence in Fujian-Taiwan Area (Co-construction), Fujian Medical University, Fuzhou, Fujian, China

**Keywords:** ANKRD1, ferroptosis, keloid, machine learning, molecular docking

## Abstract

**Background:**

Keloids are fibroproliferative skin disorders characterized by persistent extracellular matrix deposition and limited treatment options. Ferroptosis has been implicated in fibrosis, but the molecular basis of ferroptosis-related changes in keloids remains unclear.

**Methods:**

Fibroblast-derived bulk transcriptomic datasets (GSE44270 and GSE145725) were integrated to identify differentially expressed ferroptosis-related genes. Weighted gene co-expression network analysis and machine-learning algorithms were used to identify candidate hub genes, which were evaluated in an external fibroblast dataset (GSE7890). Whole-tissue single-cell RNA-sequencing data (GSE191067) were used to characterize fibroblast and myeloid-cell states in keloid and normal-skin samples. Candidate regulatory relationships and potential ANKRD1-binding compounds were assessed in silico. Clinical tissue specimens were used for histological and protein-expression validation.

**Results:**

A total of 163 differentially expressed genes were identified, and integration with 1,318 ferroptosis-related genes prioritized ANKRD1 as a candidate hub gene. ANKRD1 showed an area under the receiver operating characteristic curve above 0.80 in the external validation dataset and was positively associated with YAP1 expression in fibroblast-derived bulk transcriptomic data. Single-cell analysis identified a ECM-remodeling fibroblast state and three myeloid-cell states: inflammatory macrophages, resident-phagocytic macrophages, and DC-like myeloid cells. The ceRNA analysis identified a putative NEAT1/MALAT1-miR-493-5p-ANKRD1 regulatory axis. Molecular docking, molecular-dynamics simulation, and ADMET profiling prioritized oleanolic acid and quercetin as candidate ANKRD1-binding compounds. Histological analyses showed excessive collagen deposition and increased ANKRD1 protein expression in keloid tissues.

**Conclusion:**

ANKRD1 is a ferroptosis-related, fibroblast-associated candidate gene in keloids. The single-cell data describe fibroblast and myeloid-cell states in the keloid microenvironment. The proposed regulatory axis, candidate compounds, and potential role of ANKRD1 in fibroblast-myeloid interactions require further spatial and functional validation.

## 1 Introduction

Keloids are pathological scars that result from aberrant wound healing and are characterized by persistent fibroblast activation, dysregulated collagen turnover, and excessive extracellular matrix (ECM) deposition (1, 2). Exhibiting biological behaviors reminiscent of benign tumors, keloids grow progressively beyond the original wound margins, rarely regress spontaneously, and demonstrate notoriously high recurrence rates following surgical excision. Consequently, patients frequently endure significant physical and psychological distress, including persistent pruritus, pain, contractures, and cosmetic disfigurement, which collectively impose a substantial burden on global public health. Despite continuous advancements in multimodal treatments (1, 3–5), the development of highly efficacious targeted therapies remains hindered by the complex, highly heterogeneous pathophysiological mechanisms driving keloid formation. Therefore, elucidating the core molecular drivers of keloid fibrosis is of paramount clinical importance.

Ferroptosis, an iron-dependent form of regulated cell death driven by lipid peroxidation, has been implicated in keloid pathobiology (6, 7). The evidence indicates that keloid fibroblasts exhibit iron overload, lipid peroxidation, and depressed SLC7A11/GPX4/Nrf2 signaling, with ferroptosis inhibition attenuating fibrosis (8, 9). In contrast, ferroptosis-modulating interventions, including RSL3, deferoxamine, and 5-aminolevulinic acid photodynamic therapy, have shown context-dependent antifibrotic effects in experimental settings (10–12). These observations suggest that ferroptosis-related processes may vary across cellular compartments and experimental contexts within keloid tissue.

ANKRD1 is a mechanically responsive transcriptional cofactor that has been implicated in mesenchymal-cell activation and matrix-related processes (13). Altered ANKRD1 expression has also been reported in keloid fibroblasts, although its functional implications remain uncertain (13). Differences in sample source, culture conditions, analytical platforms, and disease context may contribute to the variable findings reported across studies. It therefore remains unclear whether ANKRD1 is associated with ferroptosis-related transcriptional changes in keloid fibroblasts and how these observations relate to the cellular composition of keloid tissue.

In this study, we integrated fibroblast-derived bulk transcriptomic datasets, weighted gene co-expression network analysis, and machine-learning-based prioritization to identify ferroptosis-related candidate genes associated with keloid fibroblast transcriptomes. ANKRD1 was selected for further analysis. Whole-tissue single-cell RNA sequencing was used to describe fibroblast and myeloid-cell states in keloid and normal-skin tissues. We also generated putative upstream regulatory relationships and computationally prioritized candidate compounds associated with ANKRD1. Finally, clinical tissue staining was used to assess collagen deposition and the immunoreactivity of ANKRD1, GPX4, and YAP. Together, these analyses provide a framework for examining ANKRD1-associated molecular features in keloids.

## 2 Materials and methods

### 2.1 Data acquisition and differential expression analysis

The Gene Expression Omnibus (GEO) is a database created by the NCBI that stores a large amount of gene expression data. Two fibroblast-derived bulk transcriptomic datasets, GSE44270 (14) and GSE145725 (15), from the Gene Expression Omnibus (GEO) database (https://www.ncbi.nlm.nih.gov/geo/) were used as the keloid transcriptome training set, and one dataset (GSE7890) (16) as the external validation transcriptome dataset. Additionally, whole-tissue single-cell RNA-sequencing data from GSE191067 (Available from: https://www.ncbi.nlm.nih.gov/geo/query/acc.cgi?acc=GSE191067.) were used to characterize the cellular landscape of keloid and normal-skin tissues. GSE44270 included 9 keloid-derived fibroblast samples and 3 control fibroblast samples. GSE145725 included 9 keloid-derived fibroblast samples and 10 control fibroblast samples. GSE7890 included 5 untreated keloid-derived fibroblast samples and 5 untreated control fibroblast samples. These datasets were used to assess fibroblast-associated transcriptional patterns. These datasets are publicly available and accessible, and additionally contain matrix information that can be well-normalized. The microarray expression data were normalized and log2-transformed before downstream analysis. The study workflow is illustrated in **Figure 1**. Detailed information is provided in **Table 1**.

**Figure 1:**
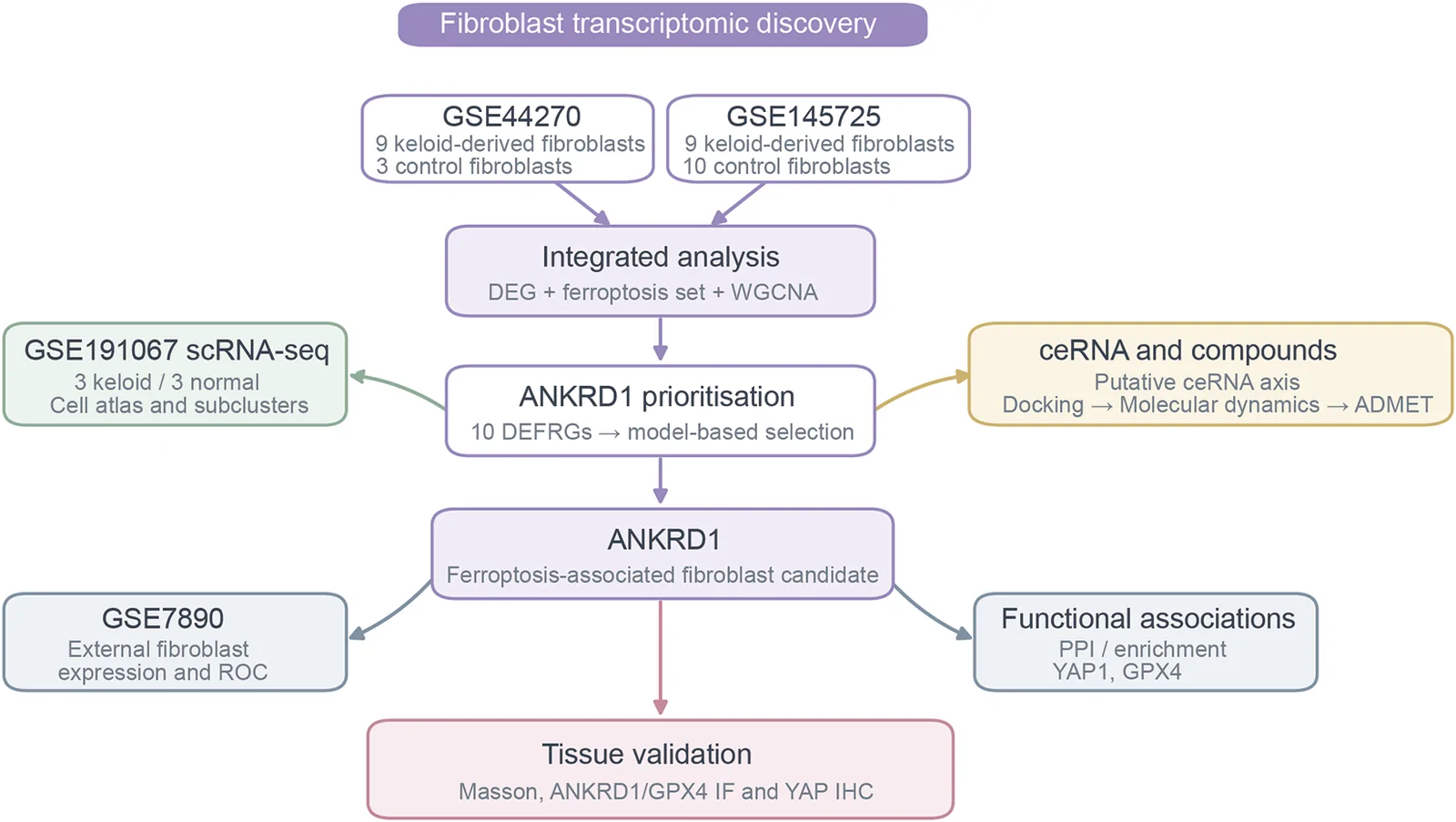
Flow chart of the study design.

**Table 1:** The basic information regarding the dataset in the present study.

Dataset type	Dataset source	Annotation platform	Keloid samples	Comparator samples
Fibroblast microarray	GSE44270	GPL6244	9	3
	GSE145725	GPL16043	9	10
	GSE7890	GPL570	5	5
scRNA-seq	GSE191067	GPL24676	3	3

For the bulk transcriptomic datasets (GSE44270, GSE145725, and GSE7890), comparator samples were control fibroblast samples. For the scRNA-seq dataset GSE191067, comparator samples were normal-skin samples.

To eliminate batch effects between GSE44270 and GSE145725, the “ComBat” function in the R package “sva” was applied (17). Differentially expressed genes (DEGs) between keloid-derived and control fibroblast samples were identified using the “Limma” R package (18), with thresholds set at p < 0.05 and |log2 fold change (FC)| > 1. Volcano plots and heatmaps were generated using “ggplot2” and “ComplexHeatmap” in R.

### 2.2 Functional enrichment analysis

Gene Ontology (GO) and Kyoto Encyclopedia of Genes and Genomes (KEGG) enrichment analyses were performed using the “clusterProfiler “ R package (19) to explore the biological functions and signaling pathways of DEGs. GO analysis included molecular function (MF), biological process (BP), and cellular component (CC). A significance threshold of p < 0.05 was used. Gene set enrichment analysis (GSEA) was performed using all genes without a predefined cutoff.

### 2.3 Identification of differentially expressed ferroptosis-related genes

Ferroptosis-related genes were obtained from the GeneCards database (https://www.genecards.org/). After removing duplicates, 1,318 ferroptosis-related genes were identified based on a relevance score > 1. DEFRGs were defined as the intersection of DEGs and ferroptosis-related genes and were visualized using a Venn diagram.

### 2.4 Weighted gene co-expression network analysis

WGCNA was performed using the “WGCNA” R package (20, 21) on the merged GSE44270 and GSE145725 datasets to identify co-expression modules associated with keloids. Modules were defined using dynamic tree cutting, and module–trait relationships were analyzed. Candidate genes were identified by intersecting DEGs, ferroptosis-related genes, and genes from significant WGCNA modules.

For functional annotation of trait-associated modules, hub genes were defined as those with |module membership| ≥ 0.7 and |gene significance| ≥ 0.5. Over-representation analysis of hub genes was performed using clusterProfiler against GO BP, KEGG, Reactome, MSigDB Hallmark, and MSigDB C2 curated gene sets. For modules with sparse hub-gene enrichment, gene-set enrichment analysis was conducted using all genes ranked by gene significance. Benjamini–Hochberg FDR < 0.05 was used as the significance threshold for module–trait associations, and p < 0.05 for enrichment terms unless otherwise stated.

### 2.5 Machine learning algorithms for hub gene screening

Machine learning is widely applied in the biomedical field to efficiently and rapidly analyze biological data, enabling accurate identification of key genes within gene expression profiles (22). To further enhance the quality of identifying key differential expression genes (DEFRGs) in keloids, two machine learning algorithms were employed to identify candidate genes in the present study, namely, random forest (RF) and support vector machine recursive feature elimination (SVM-RFE). Candidate core genes were defined as the intersection of the predictive results from both algorithms. The transcriptomic classification performance of these hub genes was then assessed in the independent validation cohort (GSE7890) using Receiver Operating Characteristic (ROC) curve analysis via the pROC R package. Bootstrap validation of diagnostic performance. To assess the stability of the ANKRD1 ROC-AUC in the training cohort, we performed bootstrap resampling (n = 1,000 iterations with replacement) and leave-one-out cross-validation (LOOCV). The 95% confidence interval of the bootstrap AUC distribution was calculated as the 2.5th and 97.5th percentiles.

### 2.6 Single-cell RNA sequencing analysis

Single-cell sequencing data analysis has been applied to explore various human skin fibrosis diseases, yielding encouraging results (23). Publicly available human keloid and normal-skin single-cell RNA-sequencing data were downloaded from GEO (GSE191067), comprising three keloid and three normal-skin samples. Raw UMI count matrices were imported into R and processed with Seurat, with sample identity and disease condition retained as metadata. Seurat objects were created without initial cell or feature filters. Quality control considered the number of detected genes, total UMI counts, mitochondrial transcript proportion where available, and doublet status. Low-library and low-feature cells were removed using sample-specific thresholds defined as the larger of the protocol floor or the sample median minus three median absolute deviations, with floors of 200 detected genes and 500 UMIs per cell. No upper cutoff was applied to UMI counts or detected genes. Doublets were detected with scDblFinder v1.12.0 using samples = sample_title, processing = “normFeatures”, and nfeatures = 1000, then excluded. As the expression matrix contained Ensembl-formatted identifiers and no MT-prefixed genes were detected, mitochondrial transcript proportion was not used as a hard filtering criterion. A total of 81,245 of 89,829 cells passed quality control. Data were normalized with SCTransform v2 using glmGamPoi acceleration and 3,000 variable features. PCA was performed on the SCT assay using 50 components, followed by Harmony v2.0.5 integration with sample identity as the batch variable; disease condition was not regressed out. UMAP visualization, shared-nearest-neighbor graph construction, and Leiden clustering were based on Harmony embeddings. The number of dimensions was set to the smaller of the 90% cumulative-variance cutoff and 30; 30 dimensions were used when this rule was undefined or returned fewer than 20 dimensions. Leiden clustering used algorithm 4 and a resolution of 0.5. Cell types were assigned using canonical lineage markers and cluster-enriched genes. Cluster markers were identified with Wilcoxon rank-sum tests on SCT-normalized data using cluster-versus-rest comparisons, positive markers only, min.pct = 0.10, logfc.threshold = 0.10, and Benjamini-Hochberg adjustment. Fibroblast and myeloid populations were analyzed separately. Fibroblast markers were identified with FindAllMarkers using the Wilcoxon test, SCT data layer, positive markers only, min.pct = 0.10, logfc.threshold = 0.10, and return.thresh = 1; clustering stability was assessed across resolutions from 0.2 to 2.0, and resolution 0.4 was used for the final fibroblast subgroup summary. Myeloid-cell identity programs were summarized using curated marker-gene sets representing resident-phagocytic, inflammatory, and antigen-presenting features. For visualization, mean log-normalized expression was calculated for each program within each annotated myeloid state and displayed as row-wise Z-scores. These program summaries were used to support cell-state annotation and were not used to infer donor-level differences in macrophage polarization between conditions. Functional enrichment used GO Biological Process and curated pathway gene sets with an expressed-gene background. Exploratory hallmark enrichment used fgsea with minSize = 10, maxSize = 500, and nperm = 10000; P values were adjusted using the Benjamini-Hochberg method.

### 2.7 Gene regulatory network

To identify regulatory factors linked to the core gene, miRNAs targeting ANKRD1 were predicted using five miRNA–target resources (miRTarBase, StarBase, miRDB, miRWalk and TargetScan) (24, 25). The predicted hsa-miR-493-5p binding site within the ANKRD1 3′UTR (ENST00000371697.3) was examined at the sequence level, and seed-pairing class was annotated according to TargetScan conventions. Upstream lncRNAs interacting with hsa-miR-493-5p were predicted using StarBase and miRNet, and experimentally supported lncRNA–miRNA interactions were queried against AGO CLIP-seq records in starBase/ENCORI v3.0, with site class (8mer/7mer-m8) and CLIP experiment counts recorded for each candidate.

### 2.8 Molecular docking and candidate compound selection

Candidate natural compounds were selected according to three predefined criteria: (i) previously reported antifibrotic or anti-inflammatory activity in skin fibrosis, keloid or related organ fibrosis contexts; (ii) natural origin with a relatively well-documented safety profile and potential for topical or intralesional formulation; and (iii) chemical scaffold diversity (flavonoid, triterpenoid, quinone and polyphenol) to minimize chemotype bias. Four compounds met these criteria: resveratrol, quercetin, tanshinone IIA and oleanolic acid. The three-dimensional structure of human ANKRD1 was obtained from the AlphaFold Protein Structure Database (entry AF-Q15327) via UniProt and preprocessed using AutoDockTools (version 1.5.6). Molecular structures of the four compounds were retrieved from the PubChem database (https://pubchem.ncbi.nlm.nih.gov/). Molecular docking was performed using CB-DOCK2 (https://cadd.labshare.cn/cb-dock2/php/index.php) with default parameters. Docking results were visualized as heatmaps using the ‘pheatmap’ package in R.

### 2.9 Molecular dynamics simulation

Molecular dynamics (MD) simulations were performed to compare the dynamic behavior of ANKRD1 complexes with quercetin, oleanolic acid, and tanshinone IIA. The ANKRD1 protein was described using the AMBER99SB-ILDN force field, and ligand topologies were generated using GAFF2 parameters with AM1-BCC partial charges. Each complex was solvated in a TIP3P water box, neutralized, and supplemented with 0.15 M NaCl. Following energy minimization, each system was equilibrated under position-restrained NVT and NPT conditions for 100 ps each. Production simulations were performed for 100 ns using GROMACS 2025.4 with a 2 fs integration time step. Long-range electrostatic interactions were treated using the particle mesh Ewald method, and bonds involving hydrogen atoms were constrained using the LINCS algorithm.

### 2.10 Trajectory analysis

Before trajectory analysis, periodic boundary conditions were corrected and each trajectory was centered on the protein. Protein backbone root-mean-square deviation (RMSD), protein radius of gyration (Rg), protein–ligand minimum distance, and protein–ligand atom-pair contacts within 0.6 nm were calculated over the 100 ns simulations. Ligand RMSD after protein-backbone fitting was calculated to assess ligand positional and conformational rearrangement relative to the protein. These trajectory descriptors were interpreted jointly to describe protein conformational behavior and ligand proximity. Structural snapshots at 0, 50, and 100 ns were generated to visualize representative conformations. As one trajectory was generated for each complex, MD results were interpreted descriptively and were used for computational prioritization rather than statistical comparison of binding stability.

### 2.11 ADMET prediction

The pharmacokinetic and drug-likeness profiles of the four candidate compounds were predicted using SwissADME (http://www.swissadme.ch/), including gastrointestinal absorption, skin permeation coefficient (Log Kp), bioavailability score, and compliance with five major drug-likeness rules (Lipinski, Ghose, Veber, Egan and Muegge). Mutagenicity (AMES test) and hepatotoxicity were additionally queried using pkCSM (https://biosig.lab.uq.edu.au/pkcsm/). Given that topical and intralesional administration represent the mainstream delivery routes for keloid pharmacotherapy, the skin permeation coefficient was prioritized over oral pharmacokinetic parameters in candidate ranking.

### 2.12 Clinical sample acquisition

This study was approved by the Ethics Committee of our hospital (approval No. 2022-431) and was conducted in accordance with the 2013 revised Declaration of Helsinki. Ethical approval was obtained prior to the initiation of sample collection and remained valid throughout the entire study period. Human tissue samples were collected between December 2024 and December 2025, a period fully covered by the approved ethical protocol. All samples were obtained as residual tissues from routine surgical procedures. Specifically, mature keloid samples were collected from patients undergoing surgical resection of keloids, while normal skin samples were obtained as redundant normal skin tissue adjacent to the resected keloids (commonly referred to as “dog-ear” tissue). A total of four keloid and four normal skin samples were included. Written informed consent was obtained from each participant prior to tissue collection. All procedures were performed in accordance with institutional guidelines.

### 2.13 Hematoxylin and eosin staining

Paraffin-embedded keloid and adjacent normal-skin sections were dewaxed, rehydrated, stained with hematoxylin for 3 min, briefly differentiated in 95% ethanol, counterstained with eosin for 15 s, dehydrated through graded alcohols, cleared with xylene, and mounted with neutral resin. Histological morphology was examined using a Nikon light microscope.

### 2.14 Masson’s trichrome staining

Collagen deposition was assessed using Masson’s trichrome staining. Paraffin sections were dewaxed, rehydrated, mordanted overnight in 2.5% potassium dichromate, and sequentially stained with Weigert’s iron hematoxylin (1 min), Ponceau-acid fuchsin (6 min), phosphomolybdic acid (1 min), and aniline blue (2–30 s). Sections were dehydrated through anhydrous ethanol, cleared with xylene, mounted with neutral resin, and examined using a Nikon light microscope. For quantitative analysis, five non-overlapping dermal fields (×20) were selected from each specimen. Color deconvolution in Fiji (ImageJ) was used to isolate the collagen-positive blue channel, and a prespecified fixed threshold was applied uniformly to segment collagen-positive areas. Collagen area fraction was calculated as the percentage of collagen-positive area within each field. The mean value across the five fields was used as the specimen-level measurement.

### 2.15 Immunohistochemistry

Formalin-fixed, paraffin-embedded keloid and adjacent normal-skin sections were dewaxed, rehydrated, subjected to heat-induced antigen retrieval, and blocked with normal serum. Sections were incubated overnight at 4 °C with rabbit anti-YAP antibody (1:50; Servicebio, China). After washing three times with phosphate-buffered saline, sections were incubated with an HRP-conjugated secondary antibody for 50 min at room temperature, developed with DAB, counterstained with hematoxylin, and imaged using a Nikon light microscope. For semi-quantitative analysis, five non-overlapping dermal fields (×10) were evaluated for each specimen. Nuclear YAP immunoreactivity was assessed using an H-score, calculated as Σ (staining-intensity category [0–3] × percentage of cells in that category), yielding a score from 0 to 300. The mean H-score across the five fields was used as the specimen-level measurement.

### 2.16 Immunofluorescence

Paraffin sections were dewaxed, rehydrated, and blocked with bovine serum albumin for 30 min. Sections were incubated overnight at 4 °C with mouse anti-ANKRD1 antibody (1:2,000; Sanying, China) or mouse anti-GPX4 antibody (1:300; Servicebio, China). After washing, sections were incubated with CY3-conjugated goat anti-mouse IgG (1:300; Servicebio, China) for 50 min at room temperature. Nuclei were counterstained with DAPI (Servicebio, China). Images were acquired using a Nikon fluorescence microscope. For semi-quantitative analysis, five non-overlapping dermal fields (×10) were selected from each specimen. Integrated optical density (IOD) was quantified within manually delineated positive dermal regions using Fiji (ImageJ) and used as a semi-quantitative measure of ANKRD1 or GPX4 immunoreactivity. The mean IOD across the five fields was used as the specimen-level measurement for statistical analysis.

### 2.17 Statistical analysis

For histological and immunostaining analyses, measurements from five image fields were averaged to obtain one value per specimen, and the specimen was treated as the biological unit for statistical analysis. All statistical analyses were performed using R (version 4.2.1) and the Sangerbox online platform. Comparisons between independent keloid and normal-skin specimens were analyzed using the two-sided Wilcoxon rank-sum test. Pearson and Spearman correlation coefficients were calculated between ANKRD1 expression and a panel of fibrosis/ECM-related genes (COL1A1, COL3A1, FN1, TGFBI, ACTA2, POSTN, CCN2), ferroptosis regulators (GPX4, SLC7A11, FTH1, NCOA4, ACSL4, PTGS2, SLC40A1, LPCAT3), and the mechanotransduction factor YAP1. Scatter plots with linear regression lines were generated to visualize significant associations.

## 3 Results

### 3.1 Histological characterization of keloids and normal skin

HE staining demonstrated clear morphological differences between keloids and normal skin tissue. In keloids sections, abundant fibroblasts with copious cytoplasm and prominent nucleoli were evident. Collagen fiber bundles appeared coarse, densely packed, and disorganized, with a prominent inflammatory infiltrate. In contrast, normal skin tissue exhibited well-differentiated epidermal cells with distinct stratification, fibroblasts with an appropriate cytoplasm-to-nucleus ratio, and collagen fiber bundles that were slender, relatively sparse, and arranged in an orderly pattern (**Figures 2A, B**). Masson’s trichrome staining revealed that collagen fibers in normal skin were neatly arranged, dense, and slender, forming a bundled structure with uniform blue coloration, with a clearly delineated boundary between the papillary and reticular dermis. In keloid tissue, collagen fibers were markedly increased, abnormally thickened, and arranged in a highly disorganized, whorled, or nodular pattern with intense blue staining. Numerous red-stained myofibroblasts were dispersed throughout the collagen nodules, and elastic fibers were markedly reduced (**Figures 2C, D**).

**Figure 2:**
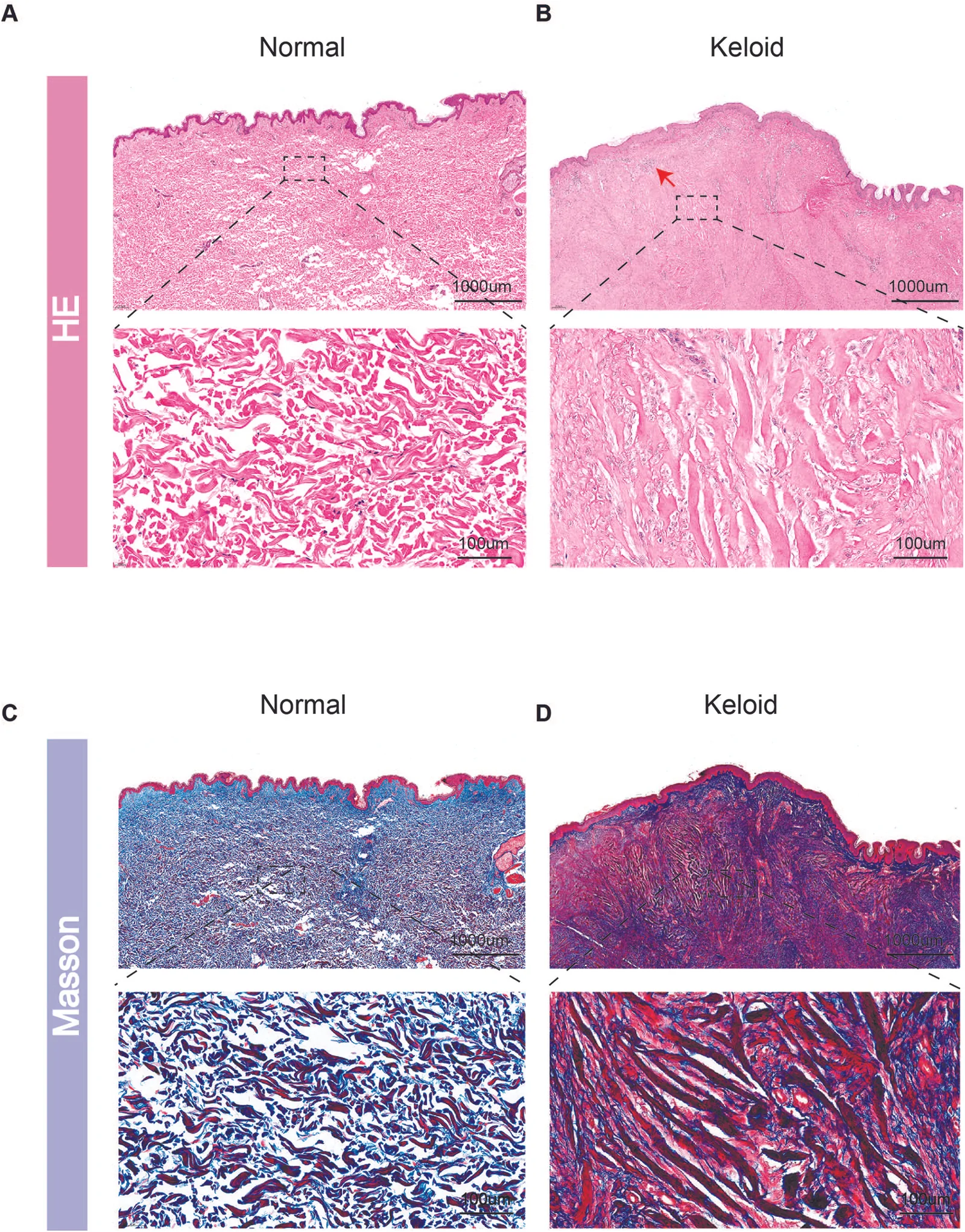
Histological characteristics and collagen deposition in normal skin and keloid tissues. **(A, B)** Representative Hematoxylin and Eosin (H&E) staining images of normal skin **(A)** and keloid tissue **(B)**. The red arrow indicates inflammatory cells. The bottom panels are magnified views of the black dashed box regions in the respective top panels. **(C, D)** Representative Masson’s trichrome staining images of normal skin **(C)** and keloid tissue **(D)**. Compared to the relatively sparse and organized collagen network in normal skin, Masson’s staining highlights the massive accumulation and altered architecture of collagen fibers (blue) in the keloid dermis. Scale bars: 1000 μm (top panels) and 100 μm (bottom panels).

### 3.2 Identification of DEGs and functional enrichment analysis

Prior to merging datasets, data distribution after normalization was evaluated (**Figure 3A**), and principal component analysis plots revealed substantial inter-dataset heterogeneity between GSE44270 and GSE145725, consistent with batch effects (**Figure 3B**). Following batch correction with the ‘ComBat’ function, sample distributions converged markedly, confirming successful batch effect removal (**Figure 3C**). A total of 163 DEGs were identified from the merged training cohort, comprising 73 upregulated and 90 downregulated genes in keloid-derived fibroblast samples relative to control fibroblast samples (all p < 0.05 and |log2FC| > 1). Differential expression patterns were visualized using a volcano plot and a heatmap (**Figure 3D**). This progressive filtering strategy prioritized a focused set of ferroptosis-related candidate genes for downstream evaluation in keloid-derived fibroblast transcriptomic datasets.

**Figure 3:**
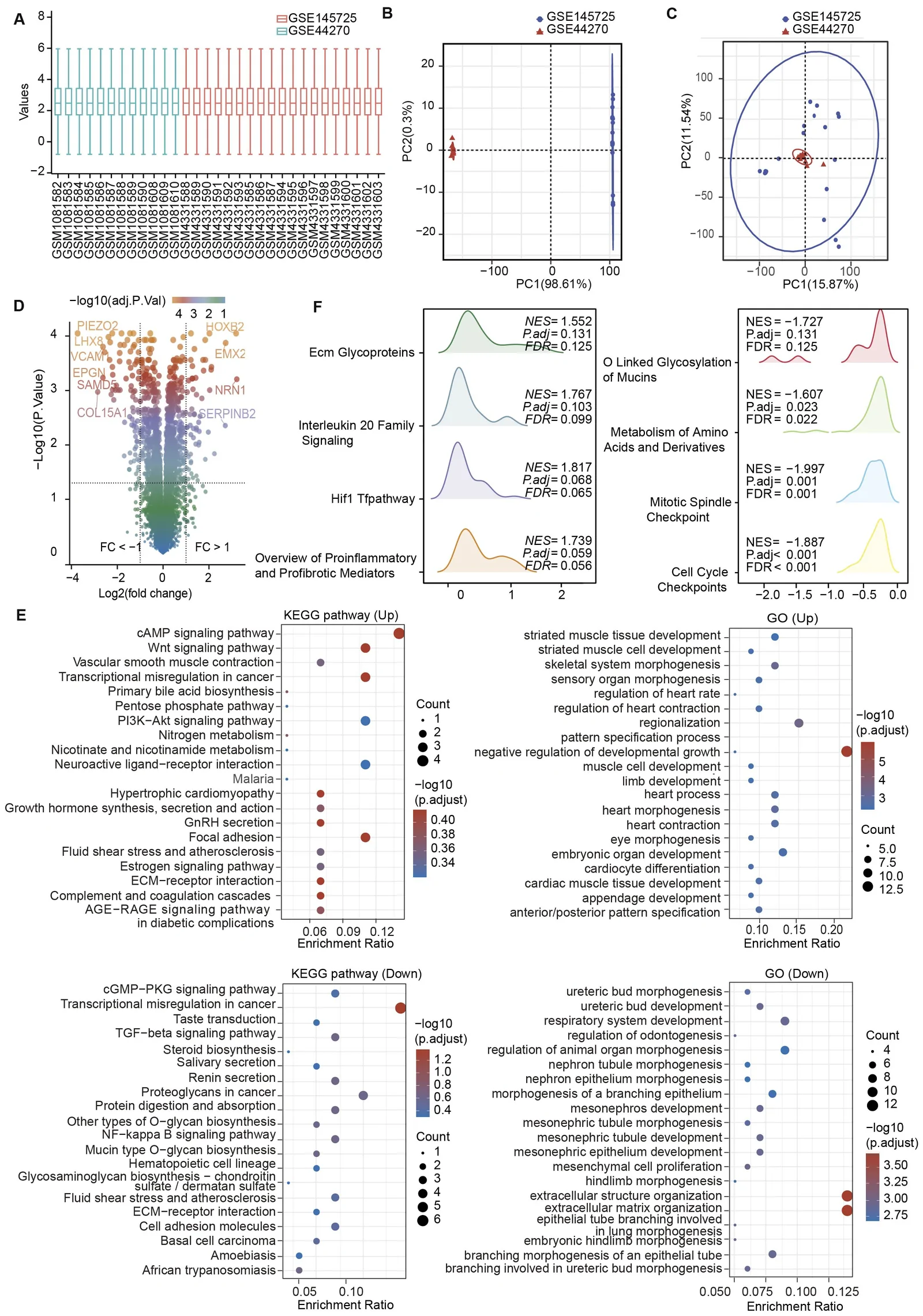
Transcriptional profiling and identification of differentially expressed genes between keloid-derived and control fibroblast samples. **(A)** Boxplots illustrating the data distribution after normalization. **(B, C)** Principal Component Analysis (PCA) of the merged datasets before **(B)** and after **(C)** the removal of batch effects. **(D)** Volcano plot highlighting DEGs between keloid-derived and control fibroblast samples. **(E)** Gene Ontology (GO) and Kyoto Encyclopedia of Genes and Genomes (KEGG) pathway analyses of the DEGs. **(F)** Gene Set Enrichment Analysis (GSEA) detailing biological pathways enriched in keloid-derived fibroblast samples.

GO analysis indicated that upregulated genes were mainly involved in “pattern specification process” and “regionalization,” while downregulated genes were primarily associated with “extracellular structure organization” and “extracellular matrix organization.” (**Figure 3E**). KEGG enrichment analysis indicated that up-regulated genes were primarily involved in pathways including “cAMP signaling pathway,” “Wnt signaling pathway,” “Transcriptional misregulation in cancer,” “PI3K-Akt signaling pathway,” “Neuroactive ligand-receptor interaction,” and “Focal adhesion.” Downregulated genes were primarily associated with “Transcriptional misregulation in cancer” and “Proteoglycans in cancer.” (**Figure 3E**). Additionally, we performed GSEA enrichment analysis. Results indicate that keloids-associated differentially expressed genes are primarily enriched in pathways including “ECM Glycoproteins,” “Overview of Proinflammatory and Profibrotic Mediators,” “Interleukin 20 Family Signaling,” “Hif1 Tfpathway,” “Mitotic Spindle Checkpoint,” and “Cell Cycle Checkpoints”. Among these, “cAMP signaling pathway,” “Wnt signaling pathway,” “PI3K-Akt signaling pathway,” “ECM Glycoproteins,” “Interleukin 20 Family Signaling,” and “Hif1 Tfpathway” align precisely with our previous understanding of keloids (**Figure 3F**).

### 3.3 Progressive filtering of ferroptosis-related hub genes via WGCNA and machine learning

To identify ferroptosis-related hub genes, a three-step filtering strategy was applied. First, WGCNA was performed on the merged training datasets using a soft-thresholding power β = 8, which achieved a scale-free topology fit index R² = 0.80 (**Figure 4A**). Dynamic tree cutting identified 24 co-expression modules (**Figure 4B**; **Supplementary Figure S1**). Module–trait correlation analysis revealed seven modules significantly associated with fibroblast source (keloid-derived versus control) after Benjamini–Hochberg FDR correction (FDR < 0.05): blue (r = 0.782, FDR = 5.02 × 10^−6^, n = 2,275), darkturquoise (r = 0.546, FDR = 0.018), cyan (r = −0.496, FDR = 0.031), grey60 (r = 0.485, FDR = 0.031), green (r = −0.479, FDR = 0.031), magenta (r = −0.438, FDR = 0.048), and tan (r = 0.437, FDR = 0.048) (**Figure 4C**). To interpret these modules functionally, hub genes (|module membership| ≥ 0.7 and |gene significance| ≥ 0.5) from each significant module were subjected to over-representation analysis (GO BP, KEGG, Reactome, Hallmark, and MSigDB C2 curated gene sets), and gene-set enrichment analysis based on gene-significance ranking was applied to modules with sparse hub-gene enrichment. The resulting functional themes were: blue – development/embryonic morphogenesis; darkturquoise – RNA metabolism/translation; cyan – inflammation/signaling (Hippo/TNF-α); grey60 – lipid/cholesterol metabolism; green – hypoxia/metabolism/EMT/glycolysis; magenta – immune chemotaxis/neutrophil chemotaxis; and tan – immune signaling (IL-6–JAK–STAT3/IFN-γ). Among the significant modules, the blue module showed the strongest positive correlation with keloid status, contained the largest number of ferroptosis-related genes (n = 141). This modular heterogeneity reinforces that keloid pathogenesis is driven by multiple, partially independent biological programs, with ANKRD1 representing one mechanosensitive node within the developmental/ferroptosis-related blue module.

**Figure 4:**
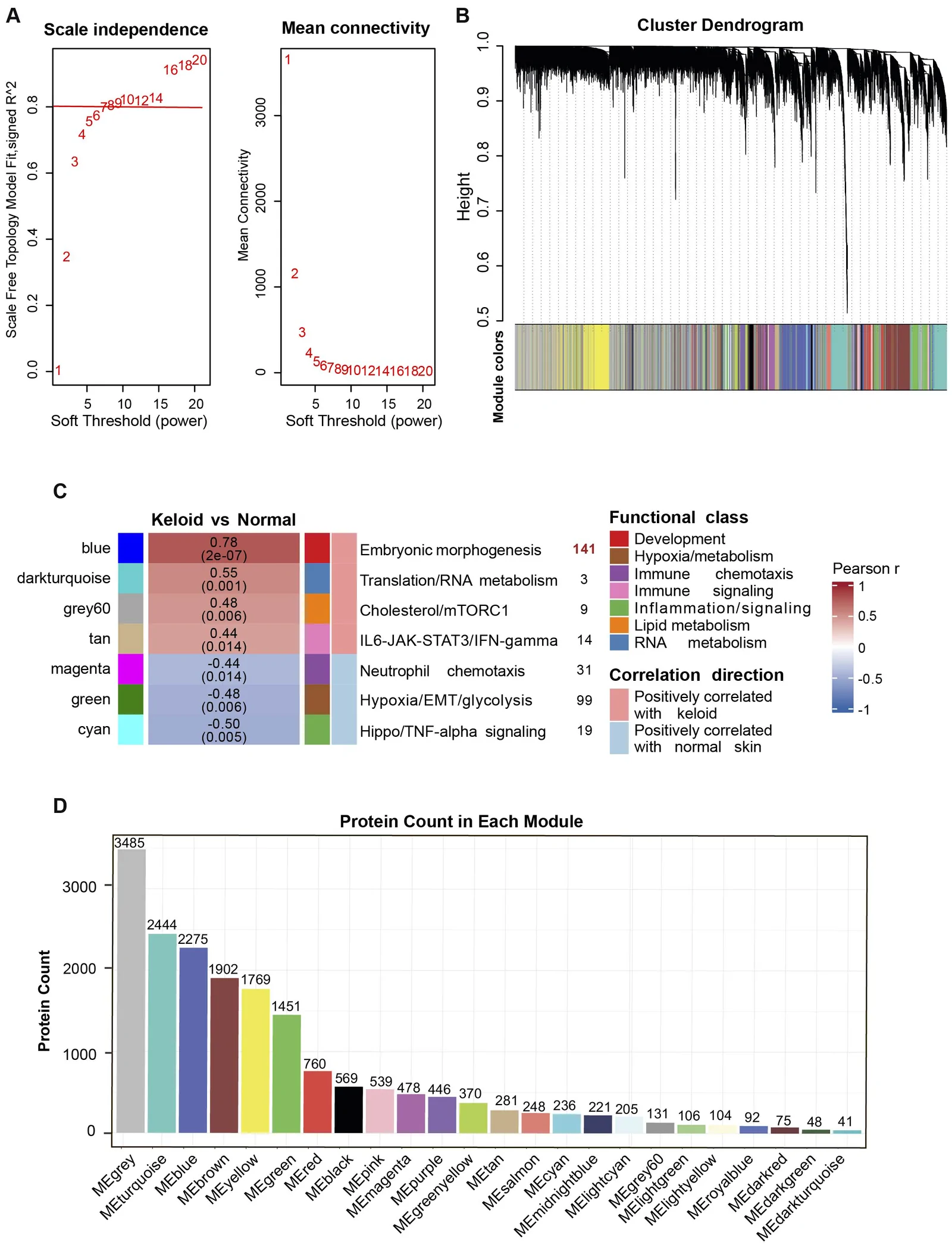
Weighted gene co-expression network analysis (WGCNA) of keloid-derived and control fibroblast transcriptomes. **(A)** Scale-free topology fit index (R²) as a function of soft-thresholding power; β = 8 was selected (R² = 0.80). **(B)** Hierarchical clustering dendrogram of all genes and the 24 co-expression modules identified by dynamic tree cutting. **(C)** Module–trait associations and functional annotations of the seven WGCNA modules significantly correlated with fibroblast source (keloid-derived versus control) (FDR < 0.05, Benjamini–Hochberg correction). The heatmap displays Pearson correlation coefficients (r) and nominal P values between module eigengenes and keloid status. Right-side annotations indicate original module colors, manually curated functional class, correlation direction, representative enriched terms, and the number of overlapping ferroptosis-related genes. **(D)** Bar plot depicting the number of proteins/genes distributed in each module.

Second, the 163 DEGs were intersected with 1,318 ferroptosis-related genes retrieved from GeneCards and with genes belonging to the seven FDR-significant WGCNA modules. This three-way intersection defined 10 differentially expressed ferroptosis-related genes (DEFRGs): STC2, DSP, ANKRD1, PITX2, RNF182, STC1, GALNT14, COL14A1, CSTA, and GPX3 (**Figures 5A, B**).

**Figure 5:**
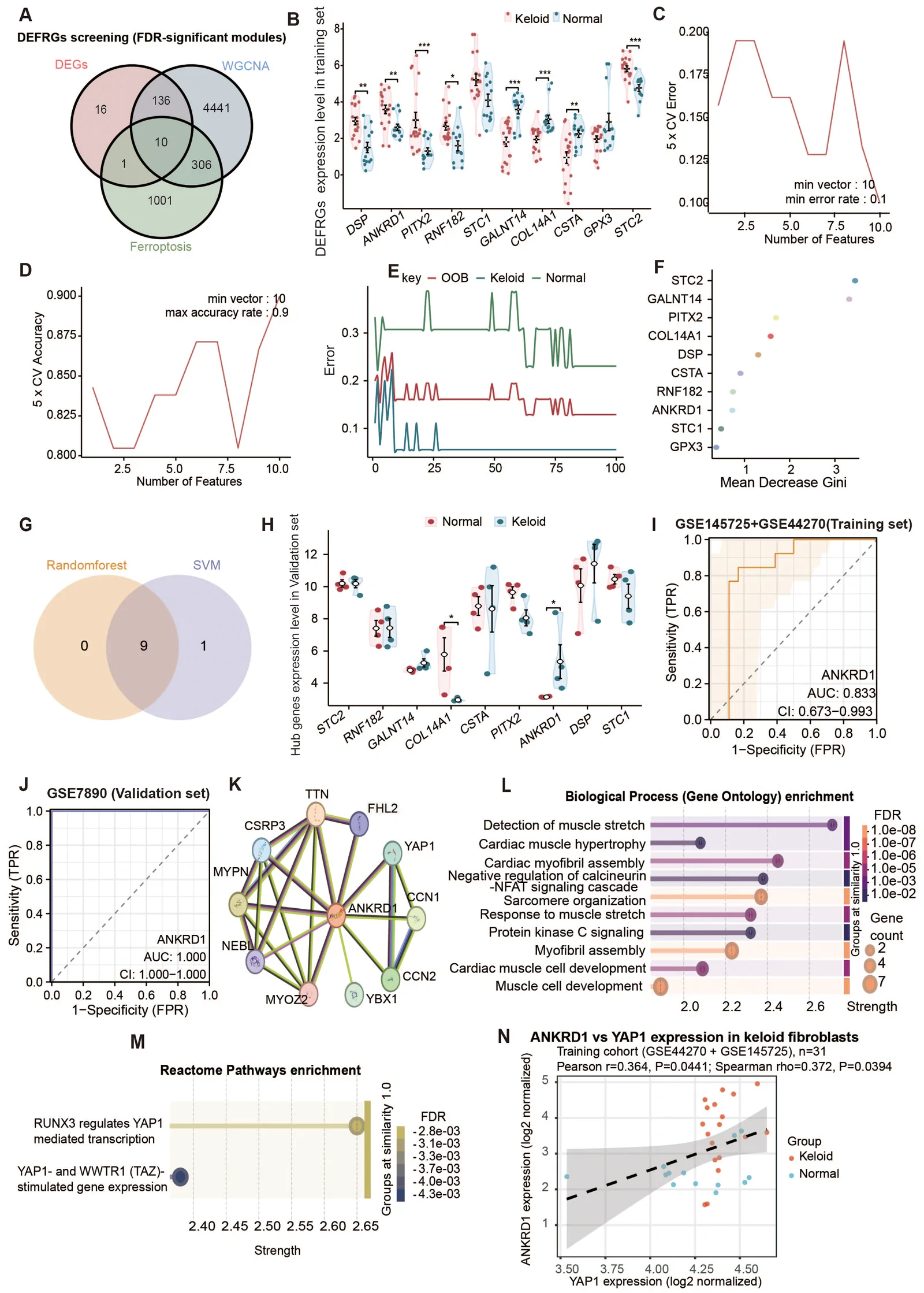
Identification and fibroblast-cohort discrimination analysis of the ferroptosis-related candidate gene ANKRD1 in keloids. **(A)** Venn diagram illustrating the intersection of DEGs, WGCNA module genes, and ferroptosis-related genes to identify 10 common ferroptosis-related DEGs (Fe-DEGs). **(B)** Expression profiles of the 10 Fe-DEGs in the training cohort. **(C, D)** Selection of hub genes utilizing the Random Forest (RF) algorithm. **(E, F)** Selection of hub genes employing the Support Vector Machine-Recursive Feature Elimination (SVM-RFE) algorithm. **(G)** Intersection of the nine consensus hub genes identified by both RF and SVM-RFE models. **(H)** Validation of ANKRD1 expression levels in an independent validation cohort. **(I, J)** Receiver operating characteristic (ROC) curves showing the ability of ANKRD1 expression to discriminate keloid-derived from control fibroblast samples in the training and external fibroblast cohorts. **(K, L)** Single-gene Gene Ontology (GO) functional enrichment analysis of ANKRD1. **(M)** Single-gene pathway enrichment analysis of ANKRD1. **(N)** Scatter plot showing the significant positive correlation between ANKRD1 and YAP1 expression in the merged training cohort (n = 31; Pearson r = 0.364, P = 0.044; Spearman rho = 0.372, P = 0.039).

Third, to further refine this candidate list, RF and SVM-RFE machine learning algorithms were independently applied to the 10 DEFRGs. The intersection of genes selected by both algorithms yielded 9 hub gene candidates—STC2, DSP, ANKRD1, PITX2, RNF182, STC1, GALNT14, COL14A1, and CSTA—representing the final set of candidate ferroptosis-associated potential biomarkers for keloid (**Figures 5C–G**).

This result suggests that genes within this module are most closely associated with keloid phenotypes and may contain key drivers of keloid pathogenesis.

### 3.4 Prioritization and fibroblast-cohort discrimination analysis of ANKRD1

Among the nine machine-learning-selected candidate genes, ANKRD1 was carried forward for further analysis because of its ferroptosis-related annotation, its selection by both machine-learning approaches, and its observed expression pattern in the available fibroblast transcriptomic cohorts. In GSE7890, ANKRD1 expression differed between keloid-derived and control fibroblast samples (**Figure 5H**). ROC analysis yielded an AUC of 0.833 in the merged training cohort and an AUC of 1.000 in GSE7890 (**Figures 5I, J**).

These ROC analyses quantify the ability of ANKRD1 expression to discriminate keloid-derived from control fibroblast microarray samples within the analyzed transcriptomic cohorts. They do not evaluate the clinical diagnosis of keloids or diagnostic performance in intact full-thickness skin tissue. In particular, the AUC of 1.000 in GSE7890 was estimated from a small cohort of five keloid-derived and five control fibroblast samples and should therefore be interpreted cautiously. Perfect separation in a small external cohort may reflect cohort-specific expression differences and does not establish reproducible diagnostic utility. Internal stratified bootstrap resampling of the merged training cohort, to match the primary analysis, yielded a mean AUC of 0.832 (95% CI, 0.654–0.970; 1,000 resamples; **Supplementary Figure S2**).

Protein–protein interaction network analysis identified proteins predicted to interact with ANKRD1 (**Figure 5K**). Functional enrichment analysis of genes associated with ANKRD1 expression highlighted mechanosensation-related terms, including “detection of muscle stretch” (**Figure 5L**), and YAP signaling (**Figure 5M**). To examine the transcriptomic relationship between ANKRD1 and YAP1, we analyzed their expression in the merged fibroblast training cohort. Across the combined cohort, ANKRD1 expression showed a modest positive association with YAP1 expression (Pearson r = 0.364, P = 0.0441; Spearman ρ = 0.372, P = 0.0394; **Figure 5N**). However, this association was not statistically significant when the analysis was restricted to keloid-derived fibroblasts (**Supplementary Figure S3**). Therefore, the correlation should be interpreted as an exploratory cohort-level observation rather than evidence of a robust within-keloid regulatory relationship. YAP is a key mechanotransduction effector and has been implicated in scar fibrosis (26), and multi-omic studies have demonstrated that mechanical activation co-upregulates YAP and ANKRD1, enriching pro-fibrotic markers and promoting pathological ECM deposition (27). Together with the enrichment results, these observations provided a rationale for prioritizing YAP-related mechanotransduction for subsequent tissue-level assessment.

### 3.5 Single-cell atlas identifies major cellular compartments in keloid and normal-skin tissues

To characterize the cellular landscape of keloid tissue, we analyzed the whole-tissue single-cell RNA-sequencing (scRNA-seq) dataset GSE191067, comprising keloid and normal-skin samples. After quality control and integration, UMAP visualization resolved 14 major cell populations, including keratinocytes, stress keratinocytes, fibroblasts, blood vascular endothelial cells, lymphatic endothelial cells, vascular mural cells, T cells, B cells, plasma cells, macrophages, mast cells, Schwann cells, sweat-gland epithelial cells, sebaceous epithelial cells, and neural cells (**Figure 6A**).

**Figure 6:**
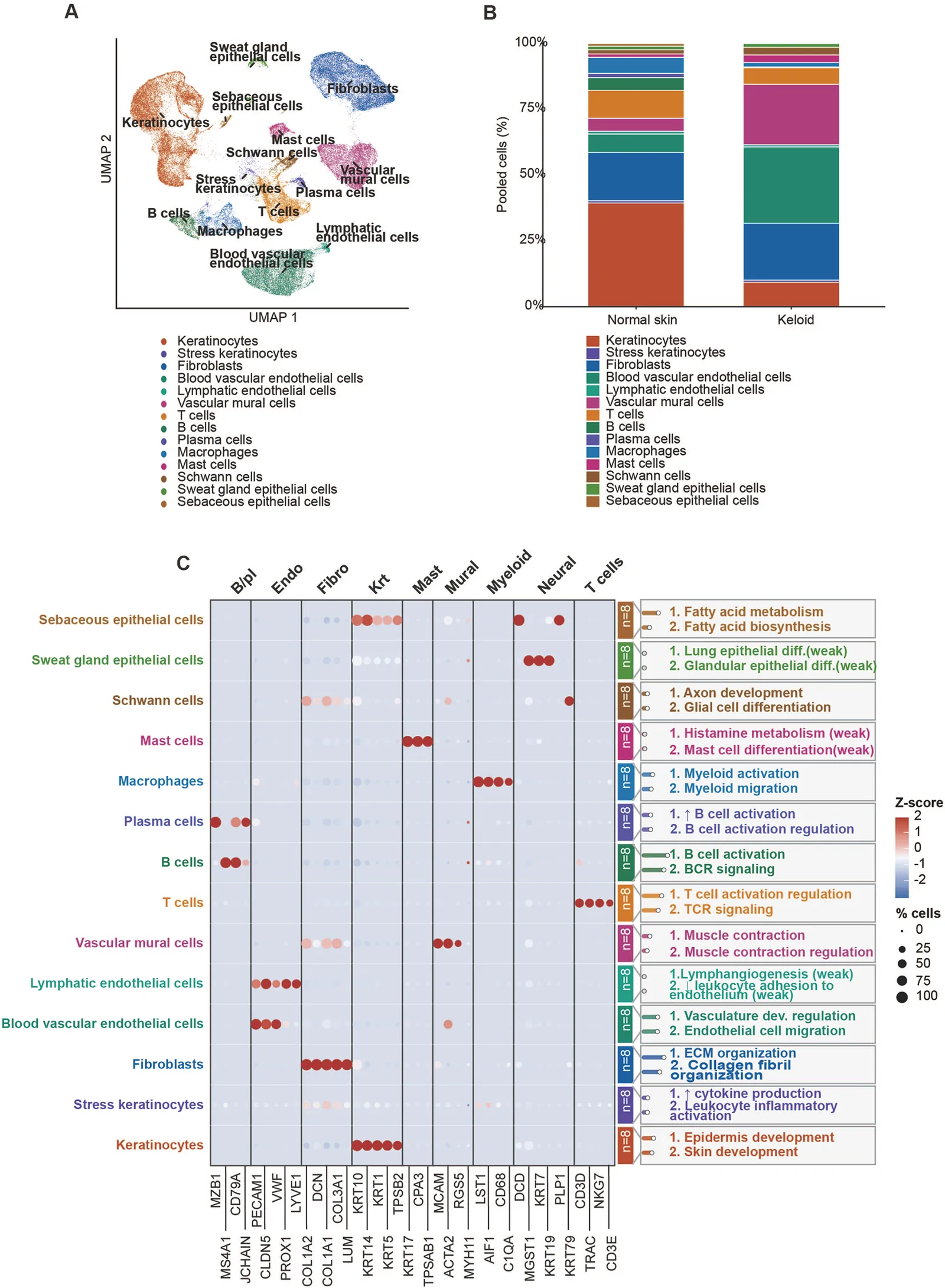
Single-cell atlas of keloid and normal-skin tissue. **(A)** UMAP visualization of the integrated single-cell dataset, colored by annotated broad cell type. **(B)** Pooled-cell composition of annotated cell populations in normal-skin and keloid samples. **(C)** Canonical marker-gene dot plot and functional annotation of broad cell populations. Dot color represents the scaled mean expression of each marker gene across the indicated cell population, and dot size represents the percentage of cells expressing the gene. Representative biological processes associated with each cell population are shown on the right, based on marker-gene functional enrichment analysis. The analysis included three keloid and three normal-skin samples from GSE191067.

The relative composition of these broad cell populations in normal-skin and keloid samples is shown in **Figure 6B**. Fibroblasts constituted a prominent cell compartment in keloid tissue, together with epithelial, vascular, and immune-cell populations. Cell identities were supported by canonical marker-gene expression and functional annotation (**Figure 6C**). Fibroblasts expressed extracellular-matrix (ECM)-associated genes, including *COL1A1*, *COL1A2*, *COL3A1*, *DCN*, and *LUM*, and were enriched for ECM organization and collagen fibril organization. Macrophages expressed myeloid markers such as *AIF1*, *CTSB*, and *CYBA* and showed enrichment of myeloid activation and migration-related programs. In parallel, lymphoid, endothelial, epithelial, and neural populations displayed lineage-consistent marker and functional signatures. This whole-tissue atlas established the cellular context for subsequent analyses of fibroblast heterogeneity and myeloid-cell states in keloid tissue.

### 3.6 An ECM-remodeling fibroblast state shows ECM- and fibrosis-associated transcriptional features

We next reclustered fibroblasts to characterize their transcriptional heterogeneity. UMAP analysis identified four fibroblast states, designated C1–C4 (**Figure 7A**). Their relative representation across individual samples is shown descriptively in **Figure 7B**. Within the fibroblast compartment, C1 showed increased expression of ECM- and fibrosis-associated genes, including COL1A1, COL3A1, COMP, ASPN, and POSTN (**Figure 7D**). Feature plots further showed the distribution of POSTN and ASPN expression within this cluster (**Figure 7C**). Gene-set enrichment analysis of C1 marker genes identified collagen fibril organization, collagen metabolic process, ECM organization, collagen biosynthetic process, connective-tissue development, wound healing, and EMT-related gene sets (**Figure 7E**). On the basis of these ECM- and fibrosis-associated transcriptional features, we designated C1 as an ECM-remodeling fibroblast state. These data describe a fibroblast state with a prominent matrix-associated transcriptional signature in the analyzed keloid dataset.

**Figure 7:**
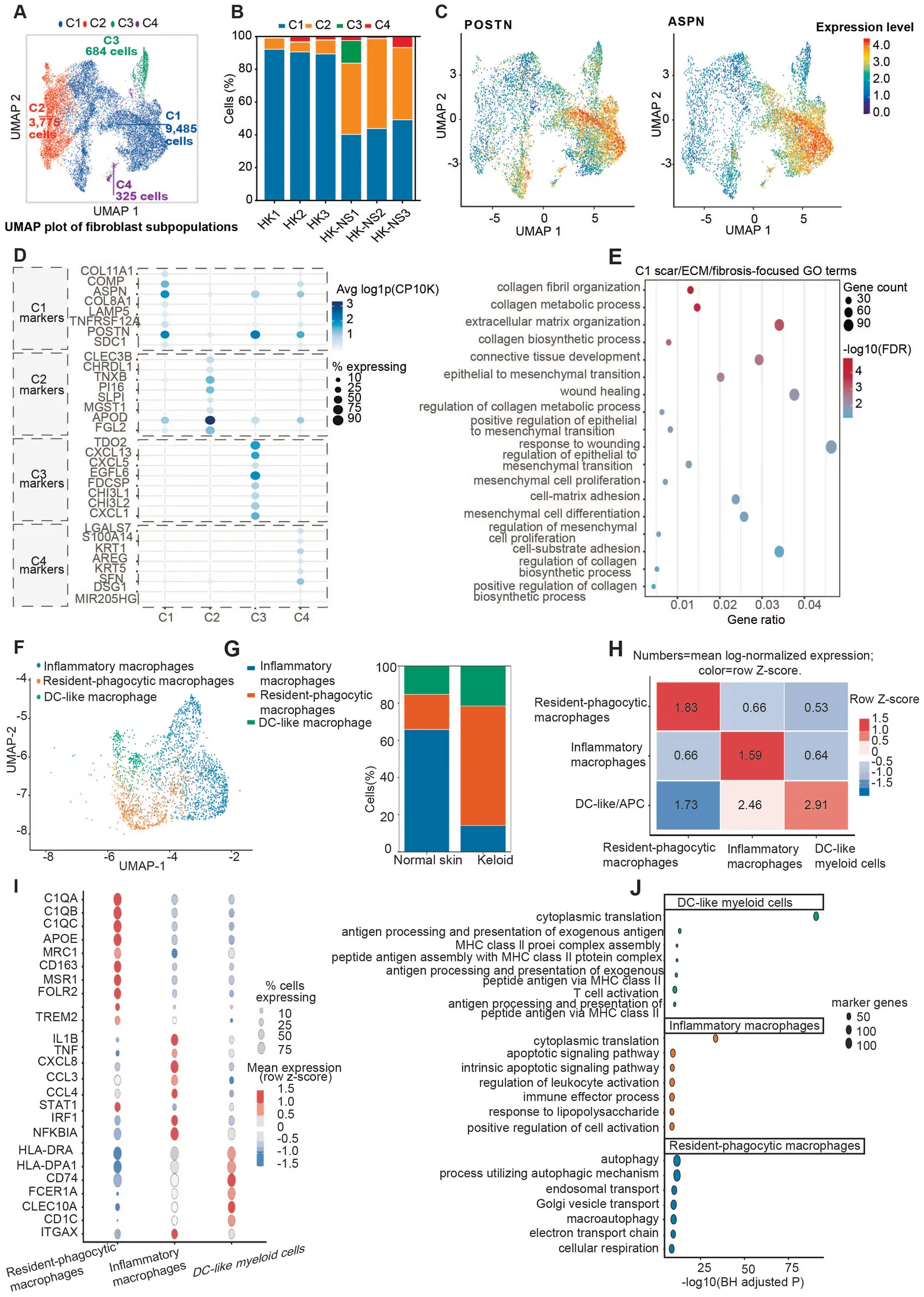
Fibroblast and macrophage/myeloid heterogeneity in keloid and normal skin. **(A)** UMAP of four fibroblast clusters (C1–C4). **(B)** Fibroblast cluster proportions across individual keloid and normal-skin samples. **(C)** UMAP feature plots showing POSTN and ASPN expression. **(D)** Dot plot of representative fibroblast markers for C1–C4. **(E)** GO enrichment of C1 markers, highlighting extracellular-matrix organization, collagen remodeling, wound healing, and fibrosis-related programs. **(F)** UMAP of macrophage/myeloid states annotated as inflammatory macrophages, resident-phagocytic macrophages, and DC-like myeloid cells. **(G)** Relative abundance of macrophage/myeloid subtypes in normal skin and keloid. **(H)** Heatmap summarizing resident-phagocytic, inflammatory, and DC-like antigen-presenting programs. **(I)** Dot plot of representative macrophage/myeloid subtype markers. **(J)** GO enrichment of subtype marker genes, showing antigen-presentation programs in DC-like myeloid cells, inflammatory/immune-effector pathways in Inflammatory macrophages, and vesicle-trafficking, autophagy, and respiratory programs in Resident-phagocytic macrophages.

### 3.7 Myeloid cells comprise inflammatory, resident-phagocytic, and antigen-presenting transcriptional states

To further characterize the myeloid compartment, macrophages and related myeloid cells were reclustered into three transcriptionally distinct states: inflammatory macrophages, resident-phagocytic macrophages, and DC-like myeloid cells (**Figure 7F**). The relative composition of these three myeloid states in normal-skin and keloid samples is shown in **Figure 7G**. The three myeloid states displayed distinct marker-gene signatures (**Figure 7I**). Resident-phagocytic macrophages expressed resident-phagocytic markers, including *C1QA*, *C1QB*, *C1QC*, *APOE*, *MRC1*, *CD163*, *MSR1*, *FOLR2*, and *TREM2*. Inflammatory macrophages were characterized by expression of inflammatory genes, including *IL1B*, *TNF*, *CXCL8*, *CCL3*, *CCL4*, *STAT1*, *IRF1*, and *NFKBIA*. DC-like myeloid cells showed expression of antigen-presentation-associated genes, including *HLA-DRA*, *HLA-DRB1*, *CD74*, *FCER1A*, *CLEC10A*, *CD1C*, and *ITGAX*. Marker-program scoring further supported the resident-phagocytic, inflammatory, and antigen-presenting features of Resident-phagocytic macrophages, Inflammatory macrophages, and DC-like myeloid cells, respectively (**Figure 7H**). Functional enrichment analysis showed that DC-like myeloid cells were associated with antigen processing and presentation pathways. Inflammatory macrophages were enriched in inflammatory signaling, leukocyte activation, and immune-effector processes, whereas Resident-phagocytic macrophages were associated with autophagy, endolysosomal transport, and cellular-respiration-related pathways (**Figure 7J**). Together, these analyses delineate a ECM-remodeling fibroblast state with pronounced ECM-remodeling activity and three transcriptionally distinct myeloid-cell states, providing a cellular framework for interpreting fibroblast-associated changes and the local immune microenvironment in keloid tissue.

### 3.8 Upstream regulatory network construction

To delineate the upstream regulatory landscape of ANKRD1, five miRNA prediction databases (miRTarBase, StarBase, miRDB, and miRWalk, TargetScan) were interrogated (**Figure 8A**). Taking the intersection across all five databases identified hsa-miR-493-5p as the only candidate shared across the five miRNA–target resources (**Figure 8B**). Sequence-level analysis of the representative ANKRD1 transcript ENST00000371697.3 (3′UTR length, 770 nt) identified a canonical 7mer-m8 candidate site at positions 729–735 (AUGUACA). This site showed complete Watson–Crick complementarity with nucleotides 2–8 of the hsa-miR-493-5p seed region, providing sequence-level support for the predicted miRNA–mRNA interaction (**Figure 8C**).

**Figure 8:**
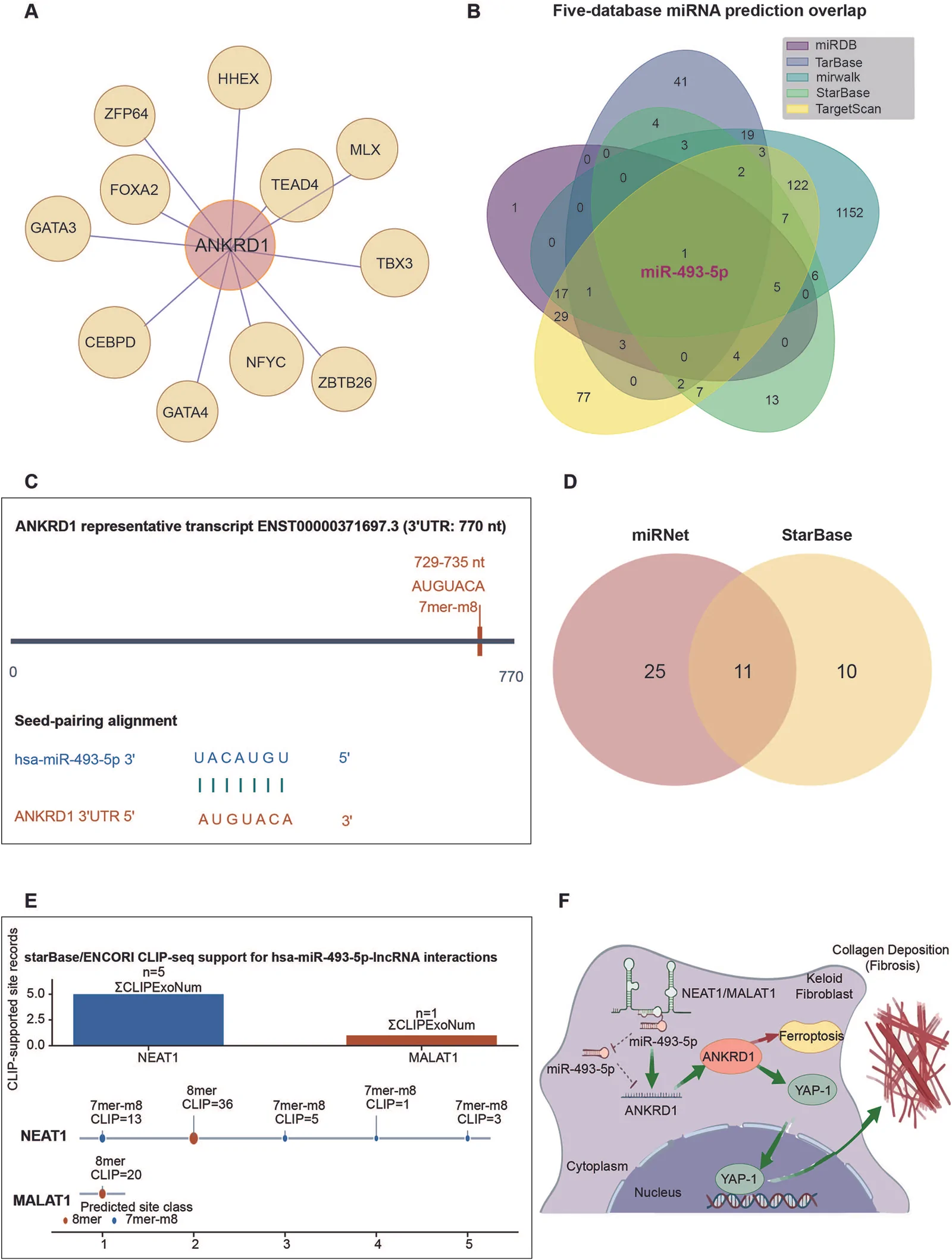
In silico identification of putative upstream regulatory relationships involving ANKRD1 in keloids. **(A)** Predicted transcriptional regulators of ANKRD1. **(B)** Overlap of candidate ANKRD1-targeting miRNAs predicted by miRDB, miRTarBase, miRWalk, StarBase, and TargetScan. hsa-miR-493-5p was consistently identified across the five databases. **(C)** Predicted seed-pairing between hsa-miR-493-5p and the 3′ untranslated region of the representative ANKRD1 transcript (ENST00000371697.3), showing a candidate 7mer-m8 site at nucleotides 729–735. **(D)** Overlap of hsa-miR-493-5p-associated lncRNAs identified using miRNet and StarBase. **(E)** StarBase/ENCORI CLIP-seq-supported candidate interaction-site records for NEAT1 and MALAT1 involving hsa-miR-493-5p; the lower panel shows the predicted seed-site classes and their positions within each lncRNA. **(F)** Working hypothesis integrating the predicted NEAT1/MALAT1–hsa-miR-493-5p–ANKRD1 regulatory relationships with mechanotransduction- and ferroptosis-related observations in keloids.

Upstream lncRNAs potentially interacting with hsa-miR-493-5p were next predicted using StarBase and miRNet, yielding eleven candidates: SNHG3, FGD5-AS1, MIR4458HG, OTUD6B-AS1, EBLN3P, KCNQ1OT1, NEAT1, MALAT1, MIR17HG, SNHG14, and LINC00667 (**Figure 8D**). Among these candidates, NEAT1 and MALAT1 were prioritized because of their established involvement in pathological scar formation, fibroblast activation, extracellular matrix deposition, and ceRNA-mediated regulation (28, 29), and primarily function via the ceRNA mechanism to modulate fibroblast proliferation, migration, apoptosis, and ECM deposition (30–32).

To further assess the lncRNA–miRNA interactions, AGO CLIP-seq records from starBase/ENCORI v3.0 were examined. NEAT1 contained five distinct CLIP-supported hsa-miR-493-5p site records (total CLIP support = 58), including one canonical 8mer site supported by 36 CLIP experiments and four 7mer-m8 sites supported by 13, 5, 1, and 3 CLIP experiments, respectively. MALAT1 contained one canonical 8mer site supported by 20 CLIP experiments (**Figure 8E**). These data indicate that both prioritized lncRNAs harbor experimentally supported AGO-associated hsa-miR-493-5p interaction sites, although their functional relevance in keloid fibroblasts remains to be established. Accordingly, we propose the NEAT1/MALAT1–hsa-miR-493-5p–ANKRD1 axis as a putative regulatory pathway governing ANKRD1 expression in keloids progression, though it awaits experimental validation. (**Figure 8F**).

### 3.9 Computational prioritization of candidate compounds associated with ANKRD1

Virtual screening of four natural compounds—selected *a priori* for reported antifibrotic activity, natural origin and scaffold diversity—was performed against ANKRD1 via molecular docking (**Figure 9A**). All four compounds exhibited binding energies below the threshold: resveratrol (−6.1 kcal/mol), quercetin (−6.5 kcal/mol), tanshinone IIA (−6.3 kcal/mol), and oleanolic acid (−6.6 kcal/mol). Because static docking scores cannot assess the temporal stability of ligand binding, the three top-ranked complexes were each subjected to a 100-ns MD simulation (**Figure 9B**). The ANKRD1–oleanolic acid complex displayed the lowest backbone RMSD, plateauing at approximately 2.5 nm after ~30 ns, compared with ~3.0–3.3 nm for quercetin and ~3.2–3.6 nm for tanshinone IIA. Consistently, oleanolic acid binding induced a compaction of ANKRD1, with the radius of gyration decreasing from ~3.8 to ~2.5 nm, whereas the protein remained in an extended conformation in the presence of tanshinone IIA (Rg ~4.2–4.6 nm); quercetin exhibited an intermediate profile. The minimum protein–ligand distance remained below ~0.25 nm throughout the simulation for both oleanolic acid and quercetin, whereas the tanshinone IIA complex underwent a dissociation event near 95 ns, with the minimum distance transiently exceeding 2.0 nm. Accordingly, the contact interface of the oleanolic acid complex expanded progressively (from ~1,500 to ~3,000 atom pairs within 0.6 nm), remained stable for quercetin (~1,000), and collapsed for tanshinone IIA in the final frames. Conformational snapshots at 0, 50 and 100 ns corroborated these metrics: oleanolic acid remained stably accommodated at the interface between the N-terminal α-helical extension and the ankyrin-repeat core, while tanshinone IIA visibly departed from the protein by 100 ns (**Figure 9C**). Of note, the relatively large backbone RMSD values across trajectories were primarily attributable to large-scale refolding of the long, flexible N-terminal α-helical extension (approximately 100 residues) onto the ankyrin-repeat core rather than to instability of the core domain itself. The MD-derived stability ranking (oleanolic acid > quercetin > tanshinone IIA) was concordant with the docking energies, and tanshinone IIA was accordingly deprioritized. ADMET profiling provided the third layer of evidence (**Figure 9D**). Among the four compounds, oleanolic acid showed the most favorable predicted skin permeation (log Kp = −3.77) and the highest predicted bioavailability score (0.85), supporting its computational prioritization for subsequent experimental assessment. Its predicted low gastrointestinal absorption and compliance with only one of five drug-likeness rules are consistent with the known physicochemical profile of pentacyclic triterpenoids and are of limited concern for topical/intralesional development. Quercetin and tanshinone IIA passed all five drug-likeness rules with high predicted GI absorption but showed inferior skin permeation (Log Kp = −7.01 and −5.02 cm/s, respectively), indicating that formulation strategies such as nanocarrier or liposomal encapsulation would be required for transdermal application. Resveratrol was additionally flagged as AMES-mutagenicity positive by pkCSM and was excluded from further consideration. Based on the docking scores, MD trajectories, and predicted ADMET properties, oleanolic acid was ranked as the leading compound and quercetin as a secondary candidate for further study in relation to ANKRD1 (**Figure 9**). These rankings are based solely on in silico analyses and require experimental validation.

**Figure 9:**
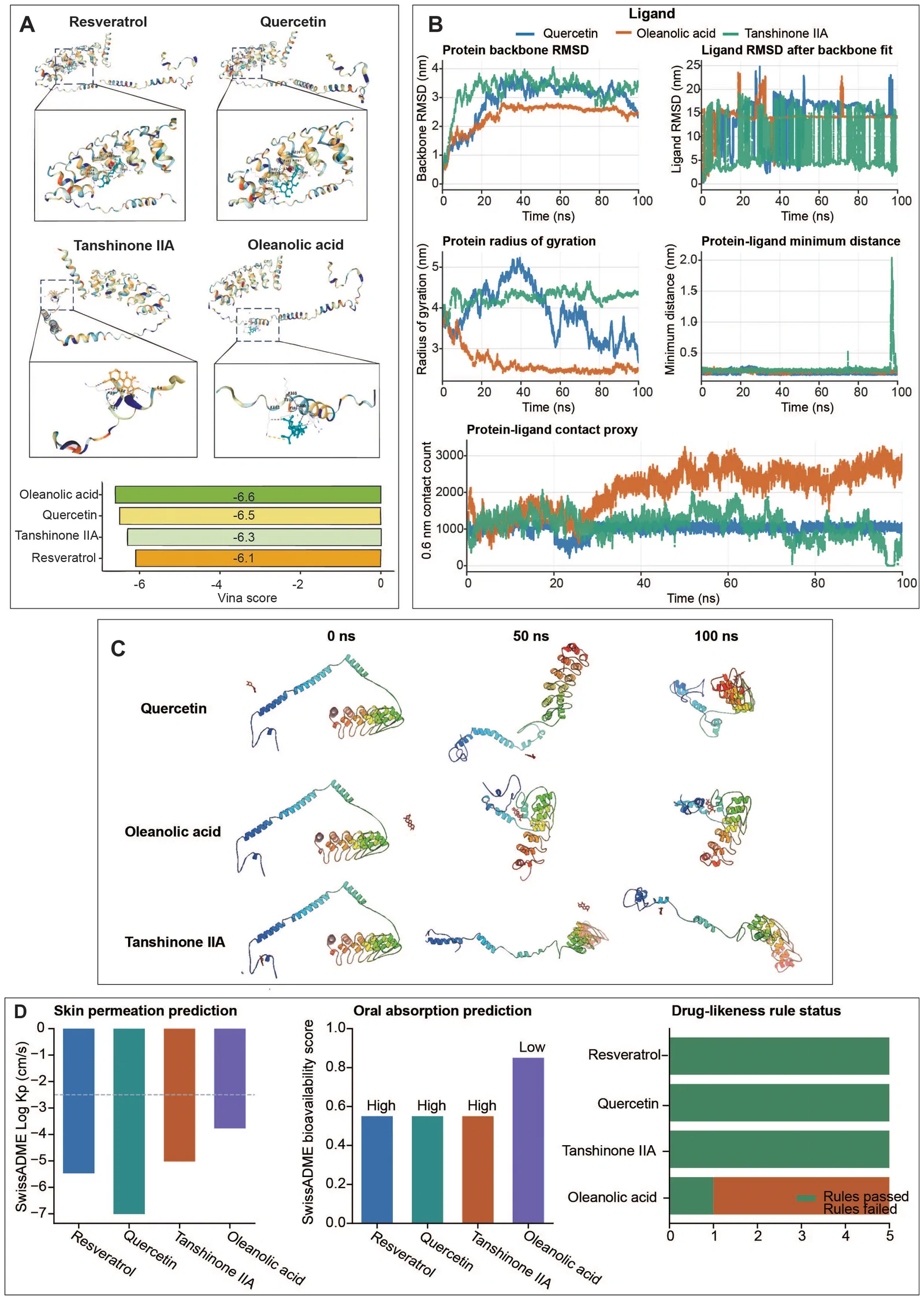
In silico assessment of candidate compounds associated with ANKRD1. **(A)** Predicted docking poses and docking scores of resveratrol, quercetin, tanshinone IIA, and oleanolic acid with the ANKRD1 structural model. **(B)** Molecular-dynamics trajectories for the ANKRD1 complexes with oleanolic acid, quercetin, and tanshinone IIA over 100 ns, including protein backbone root-mean-square deviation (RMSD), radius of gyration (Rg), minimum protein-ligand distance, and the number of protein-ligand atom pairs within a 0.6-nm cutoff. **(C)** Representative complex conformations at 0, 50, and 100 ns. **(D)** Predicted pharmacokinetic and safety-related properties of the four compounds, including gastrointestinal absorption, skin permeation coefficient (Log Kp), bioavailability score, drug-likeness-rule compliance, AMES mutagenicity, and hepatotoxicity. Docking, molecular-dynamics, and ADMET analyses were used for computational compound prioritization and do not demonstrate direct ANKRD1 binding or therapeutic activity in keloids.

### 3.10 Tissue-level assessment of ANKRD1, GPX4, and YAP expression

We performed immunohistochemical and immunofluorescence analyses on keloids and normal skin tissue samples. As a central phospholipid hydroperoxide reductase that contributes to ferroptosis defense, GPX4 plays a critical role in inhibiting ferroptosis (8, 33, 34). Immunofluorescence was therefore used to evaluate the expression levels of ANKRD1 and GPX4 in keloids and normal skin. The results showed that ANKRD1 expression was significantly upregulated in keloid tissue compared with normal skin (P <0.05) (**Figure 10A**). In contrast, GPX4 expression was significantly lower in keloids samples than in normal skin (P <0.05) (**Figure 10B**).

**Figure 10:**
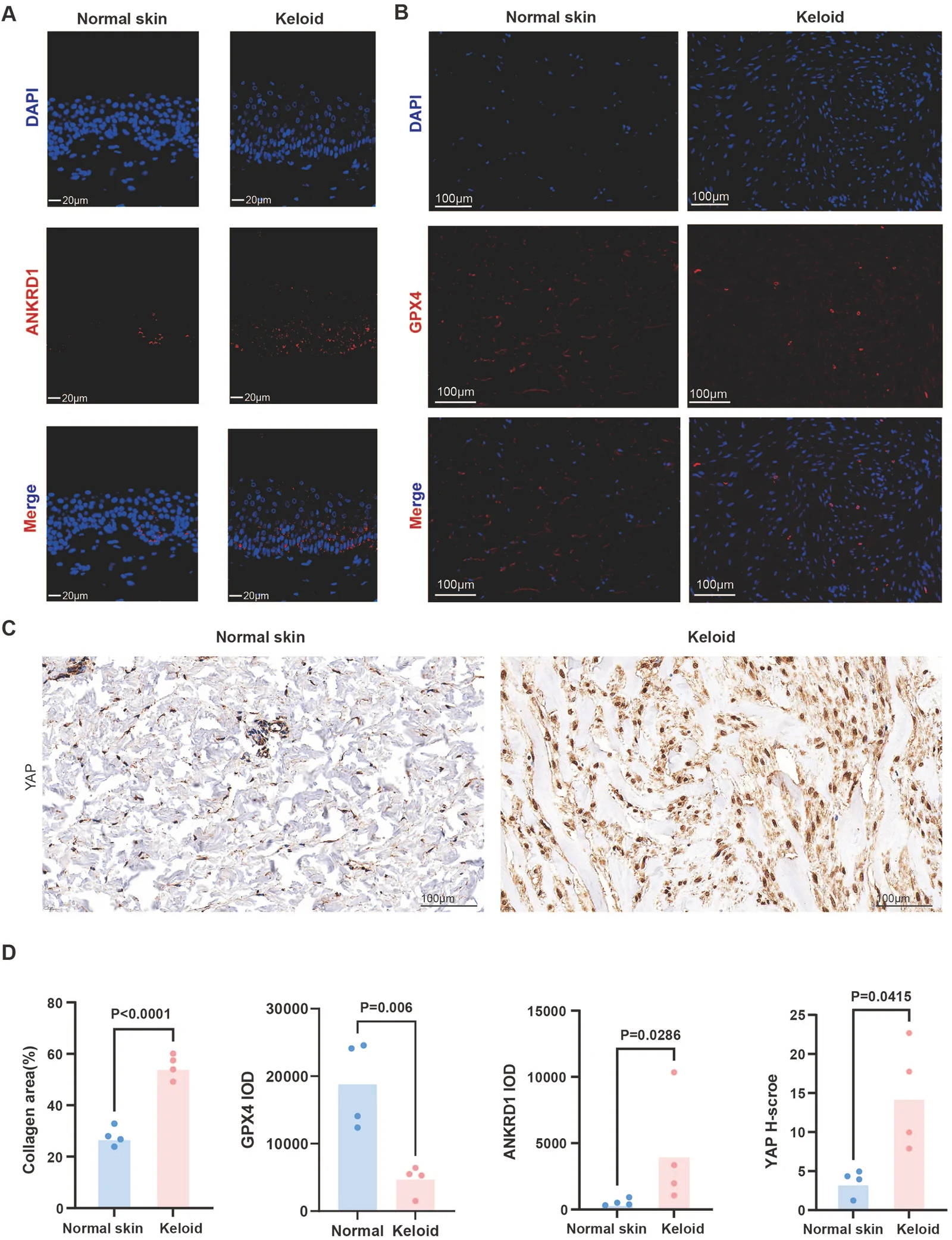
Histological and immunostaining assessment of keloid and normal-skin tissues. **(A)** Representative immunofluorescence images showing ANKRD1 (red) and DAPI-stained nuclei (blue) in normal-skin and keloid tissues. Scale bars, 20 μm. **(B)** Representative immunofluorescence images showing GPX4 (red) and DAPI-stained nuclei (blue) in normal-skin and keloid tissues. Scale bars, 100 μm. **(C)** Representative immunohistochemical staining for YAP in normal-skin and keloid tissues. Scale bars, 100 μm. **(D)** Quantification of collagen area fraction in Masson’s trichrome-stained sections, GPX4 integrated optical density (IOD), ANKRD1 IOD, and YAP H-score. Each dot represents one independent tissue specimen. Measurements from five non-overlapping dermal fields were averaged before analysis (n = 4 keloid specimens and n = 4 normal-skin specimens). Comparisons were performed using exact two-sided Wilcoxon rank-sum tests. P = 0.0286 for each comparison.

YAP immunohistochemistry showed greater nuclear YAP immunoreactivity in keloid than in normal-skin specimens. The specimen-level YAP H-score was higher in keloid specimens (P < 0.05) (**Figure 10C**). We next assessed exploratory ANKRD1-centred associations across the pooled clinical tissue cohort. ANKRD1 IOD was inversely associated with GPX4 IOD (Spearman ρ = −0.738, Holm-adjusted P = 0.046) and positively associated with collagen area fraction (ρ = 0.857, Holm-adjusted P = 0.032) and YAP H-score (ρ = 0.810, Holm-adjusted P = 0.044) (**Supplementary Figure S4**). Because these analyses pooled four keloid and four normal-skin specimens, they are interpreted as exploratory tissue-level associations and do not establish direct regulatory relationships or causality.

## 4 Discussion

Keloids are complex, fibroproliferative dermal disorders driven by aberrant wound healing, characterized by excessively high recurrence rates and a lack of universally effective treatments (35). While traditional therapies focus on surgical excision and broad-spectrum immunosuppression, the elucidation of highly specific molecular targets remains an urgent clinical need (36). In this study, we integrated fibroblast-derived bulk transcriptomic datasets, WGCNA, and machine-learning-based prioritization to identify ANKRD1 as a ferroptosis-related, fibroblast-associated candidate gene. Whole-tissue single-cell analysis further defined major cellular compartments and identified a ECM-remodeling fibroblast state with prominent extracellular-matrix and fibrosis-associated transcriptional features, together with distinct myeloid-cell states. Clinical tissue staining provided group-level evidence of altered ANKRD1, GPX4, and YAP immunoreactivity in the available specimens. Taken together, the present findings identify ANKRD1 as a fibroblast-associated candidate that correlates with ferroptosis- and mechanotransduction-related observations in keloids.

Ferroptosis, an iron-dependent form of programmed cell death driven by toxic lipid peroxidation (37), has been implicated in multiorgan fibrosis (38–41). Under physiological conditions, antioxidant systems primarily governed by GPX4 neutralize lipid peroxides and help maintain cellular redox homeostasis (42). Disturbed iron metabolism and lipid-peroxidation processes have been associated with fibroblast activation and extracellular matrix (ECM) deposition (43, 44). In experimental models, ferroptosis-related interventions have been reported to influence keloid fibroblast behavior and fibrotic phenotypes (8).Among the ferroptosis-related candidates, ANKRD1 was prioritized because it was selected by both machine-learning approaches and showed discriminatory capacity in the analyzed fibroblast cohorts. In the present study, immunofluorescence analysis showed higher ANKRD1 immunoreactivity and lower GPX4 immunoreactivity in keloid tissues than in normal-skin tissues, consistent with altered ferroptosis-related homeostasis.

Among the enriched pathways, we selected YAP signaling for further validation due to its well-established role in mechanotransduction and scar fibrosis, as well as the strong statistical signal observed in our enrichment analysis. This does not exclude the involvement of other pathways. Of particular interest, ANKRD1 is well-recognized as a mechanosensitive gene downstream of YAP signaling (45, 46), linking mechanical force responses to fibrotic progression (47). Our immunohistochemical results also revealed differential YAP immunoreactivity in keloid lesions, with increased nuclear staining in keloid tissue, implying potential crosstalk between mechanical signaling and ferroptosis in keloid pathogenesis. Mechanical overloading within keloid microenvironment may upregulate ANKRD1 via YAP activation, which could subsequently interfere with GPX4-dependent antioxidant capacity and perturb ferroptosis balance in pathogenic fibroblasts. This hypothetical axis may partially explain why keloids preferentially occur at high-tension body sites, such as the anterior chest and scapula (48, 49). Together with previous reports linking Piezo1-dependent mechanical signaling to YAP activation and matrix production in keloid fibroblasts (50, 51), these observations provided the rationale for examining YAP in relation to ANKRD1.

Beyond transcriptional regulation, post-transcriptional mechanisms may provide an additional level of ANKRD1 control. NEAT1 has been functionally implicated in keloid fibroblasts through the miR-196b-5p/FGF2 axis, and both NEAT1 and MALAT1 have been included in a predicted keloid ceRNA network (52), raising the possibility that ANKRD1 may likewise be subject to non-coding RNA-mediated regulation. However, these correlative observations are based on human tissue cross-sectional evidence; they do not establish direct YAP–ANKRD1 regulation, ANKRD1-mediated ferroptosis suppression, or the functional activity of the proposed NEAT1/MALAT1-miR-493-5p-ANKRD1 axis. Instead, ANKRD1 should be interpreted as one component of a broader keloid-associated transcriptional network rather than an isolated master regulator. Its potential relevance lies in its association with ECM-related and mechanotransduction-related features, although its relative contribution to established fibrotic pathways needs further validation in primary cells and independent tissue cohorts.

Keloid fibrosis is unlikely to reflect a uniform change across all fibroblasts. Instead, our single-cell analysis identified a ECM-remodeling fibroblast state characterized by ECM-remodeling and wound-healing-related transcriptional programs. The expression of matrix-associated genes, including POSTN and ASPN, together with the enrichment of collagen-organization pathways, suggests that this state may represent a matrix-producing fibroblast population within keloid tissue. This interpretation is consistent with previous single-cell studies showing that mesenchymal fibroblasts are expanded in keloids and contribute to collagen-associated fibrotic programs (23). More recently, POSTN-positive mesenchymal fibroblasts with prominent ECM-associated features have also been described in keloid tissue (53). Although the clustering strategies and datasets differ, these findings support the presence of mesenchymal fibroblast states associated with fibrotic matrix remodeling. The myeloid compartment also showed transcriptional heterogeneity, including inflammatory macrophage-like, resident-phagocytic macrophage-like, and DC-like myeloid states. This observation is in line with previous single-cell profiling of keloids, which identified diverse macrophage and dendritic-cell populations within the local immune microenvironment (54). Together, these findings suggest that keloid tissue contains multiple stromal and myeloid programs rather than a uniform fibroblast or macrophage phenotype. However, the present dataset was not designed to establish donor-level changes in myeloid-cell abundance or macrophage polarization. It also does not demonstrate that ANKRD1 directly determines the ECM-remodeling fibroblast state or regulates myeloid-cell behavior. Rather, the single-cell analysis provides cellular context for the fibroblast-associated ANKRD1 signal identified in the bulk transcriptomic cohorts.

Oleanolic acid and quercetin emerged from the in silico screen as compounds worth examining in keloid fibroblasts. Both have reported antifibrotic activity, and their predicted association with ANKRD1 provides a rationale for testing them in this setting. The current analysis, however, does not demonstrate direct binding to ANKRD1 or therapeutic activity in keloids. Docking, molecular-dynamics simulation, and ADMET prediction estimate structural compatibility and physicochemical properties, but cannot capture cellular target engagement or compound-specific effects in the keloid microenvironment. The molecular-dynamics trajectories suggested that ligand presence was associated with differences in the conformation of the flexible N-terminal region of ANKRD1. Whether these changes occur in cells or influence fibrotic phenotypes remains to be determined.

Several limitations should be considered. First, the ceRNA network, molecular docking, molecular-dynamics simulations, and ADMET analyses are computational and do not establish direct molecular binding, regulatory activity, or therapeutic efficacy. Second, the ROC analyses were derived from fibroblast transcriptomic cohorts and should not be interpreted as evidence of diagnostic performance in intact keloid tissue. The external fibroblast cohort was small, and larger independent fibroblast and full-thickness tissue cohorts are needed. Third, the single-cell analysis included three keloid and three normal-skin donors. It provides cellular-state information but cannot establish spatial relationships, donor-level changes in cell abundance, macrophage polarization, or ANKRD1-dependent intercellular regulation. Finally, the observed associations among ANKRD1, YAP1, GPX4, and ECM-related transcriptional features are associative. Their directionality and functional consequences require targeted perturbation experiments.

## 5 Conclusion

This study prioritizes ANKRD1 as a ferroptosis-related, fibroblast-associated candidate gene in keloids. Integrative analyses of fibroblast transcriptomic datasets linked ANKRD1 to extracellular-matrix-remodeling and mechanotransduction-related transcriptional features. Whole-tissue single-cell RNA sequencing further defined ECM-remodeling fibroblast and myeloid-cell states in keloid and normal-skin tissues. The proposed regulatory relationships and candidate compounds require validation in independent cohorts and functional studies.

## Data Availability

The original contributions presented in the study are included in the article/**Supplementary Material**. Further inquiries can be directed to the corresponding authors.

## Supplementary material

Supplementary Figure 1 Module-trait relationship heatmap. The heatmap shows correlations (coefficient and P-value) between each consensus module (ME-prefixed) and the Keloid versus Normal groups. Red indicates positive and blue negative correlation. Modules marked with a red star (*) are significant after FDR correction (adjusted P<0.05). Several modules positively correlate with Keloid, while others show significant negative correlation.

Supplementary Figure 2 Bootstrap distribution of ANKRD1 discrimination in the merged fibroblast microarray cohort. Distribution of area under the curve (AUC) values obtained from 1,000 stratified array-level bootstrap resamples of the merged GSE44270 and GSE145725 cohort. The red dashed line indicates the observed AUC of ANKRD1 (0.833), the purple solid line indicates the bootstrap mean AUC (0.832), and the grey dotted lines indicate the percentile 95% confidence interval (0.654–0.970).

Supplementary Figure 3 Exploratory association between ANKRD1 and YAP1 expression in keloid-derived fibroblasts. Scatter plot of log2-normalized ANKRD1 and YAP1 expression across keloid-derived fibroblast samples in the merged training cohort. The dashed line represents the ordinary least-squares regression fit, and the shaded area indicates the two-sided 95% confidence interval for the mean fitted relationship.

Supplementary Figure 4 Clinical tissue-level associations of ANKRD1 with GPX4, collagen burden and YAP. **(a)**, Association between ANKRD1 and GPX4 immunofluorescence intensity. **(b)**, Association between ANKRD1 immunofluorescence intensity and Masson-derived collagen area fraction. **(c)**, Association between ANKRD1 immunofluorescence intensity and YAP H-score. Each point represents one tissue specimen, including four keloid and four normal-skin specimens. Colors indicate diagnostic group. IOD values are displayed on a log10 scale for visualization; correlation testing used the original specimen-level values. Spearman correlation coefficients and exact two-sided permutation P values were calculated, with Holm adjustment across the three displayed ANKRD1-centred comparisons. Dashed lines indicate ordinary least-squares fits and shaded areas indicate 95% confidence intervals.
