# Supplementary material for: Deciphering fibroblast and myeloid-cell states in keloids: an integrative analysis identifies ANKRD1 as a ferroptosis-related candidate gene
Source: Front Immunol. 2026 Oct 7;17:1872427. doi: 10.3389/fimmu.2026.1872427 (PMC13683095; doi:10.3389/fimmu.2026.1872427)
Supplement: Supplementary file 1 [file SupplementaryFile1.pdf]

Supplementary Figure

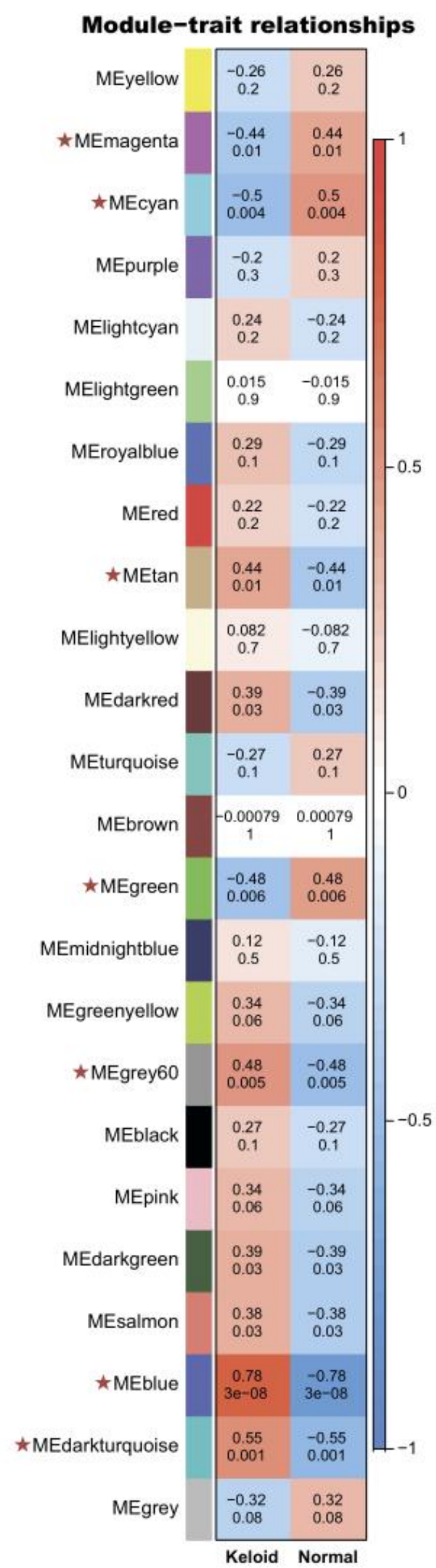

Supplementary Figure S1

Module-trait relationship heatmap. The heatmap shows correlations (coefficient and P-value) between each consensus module (ME-prefixed) and the Keloid versus Normal groups. Red indicates positive and blue negative correlation. Modules marked with a red star (★) are significant after FDR correction (adjusted P<0.05). Several modules positively correlate with Keloid, while others show significant negative correlation.

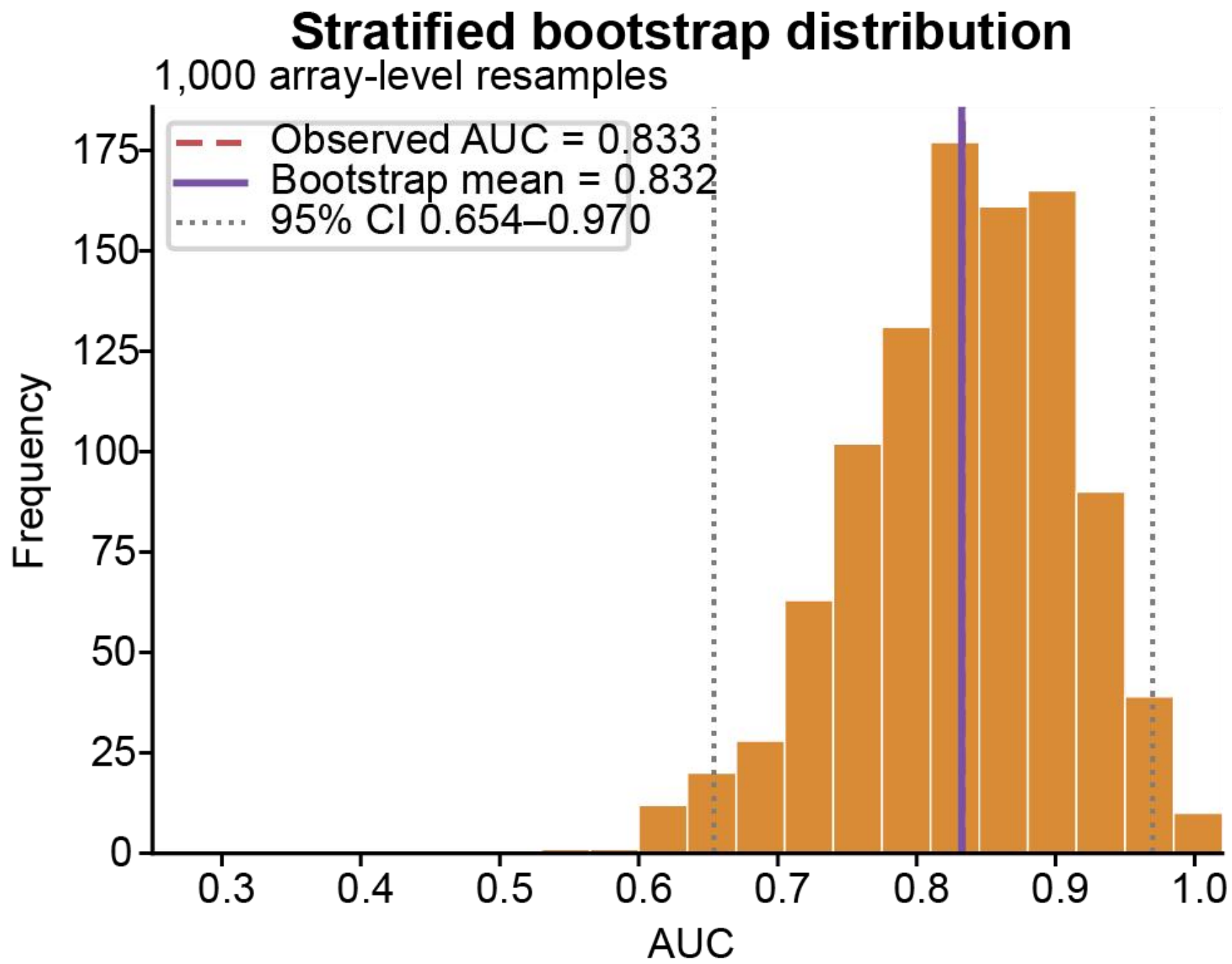

Supplementary Figure S2

Bootstrap distribution of ANKRD1 discrimination in the merged fibroblast microarray cohort. Distribution of area under the curve (AUC) values obtained from 1,000 stratified array-level bootstrap resamples of the merged GSE44270 and GSE145725 cohort. The red dashed line indicates the observed AUC of ANKRD1 (0.833), the purple solid line indicates the bootstrap mean AUC (0.832), and the grey dotted lines indicate the percentile 95% confidence interval (0.654–0.970).

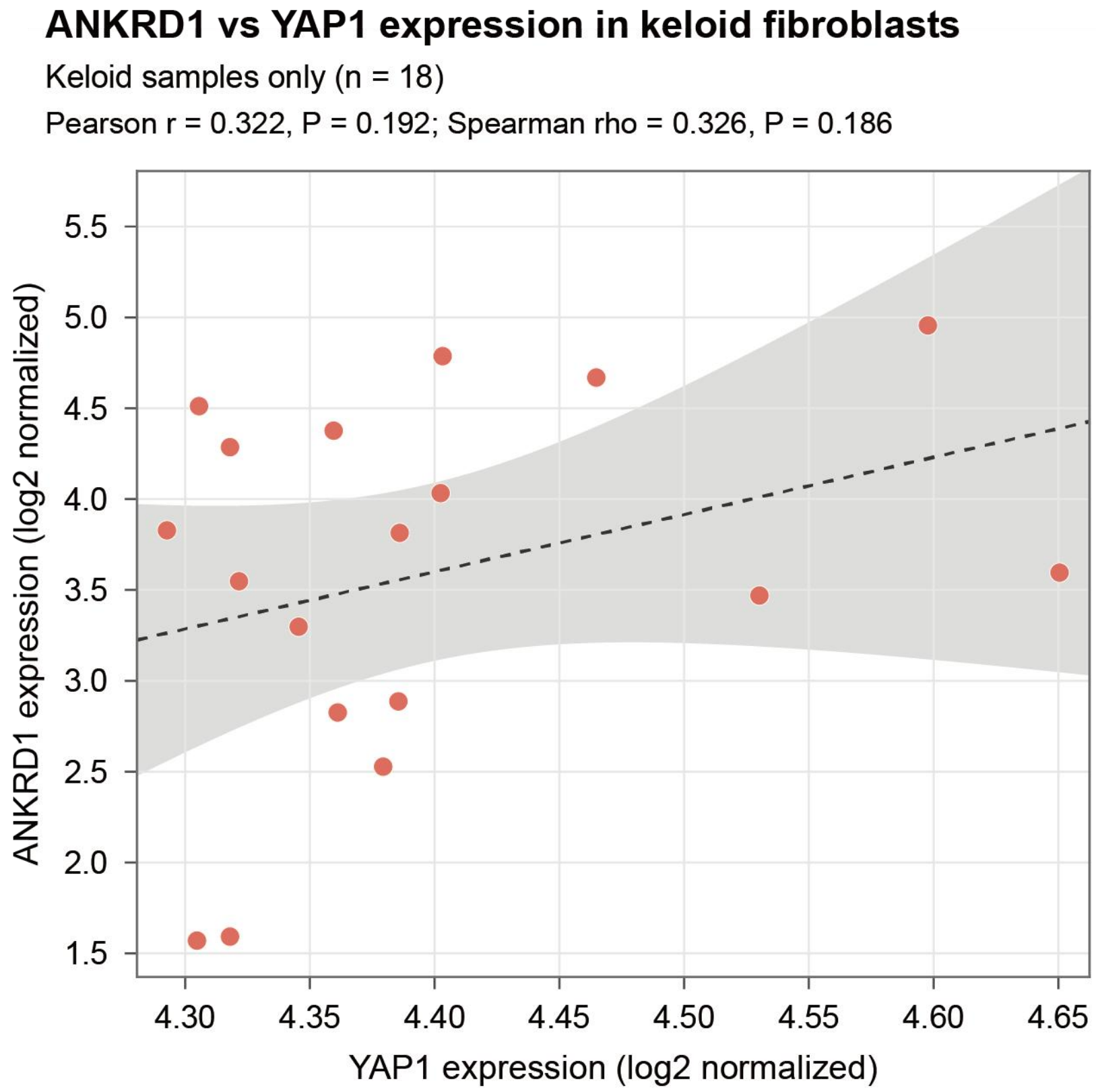

Supplementary Figure S3

Exploratory association between ANKRD1 and YAP1 expression in keloid-derived fibroblasts. Scatter plot of log2-normalized ANKRD1 and YAP1 expression across keloid-derived fibroblast samples in the merged training cohort. The dashed line represents the ordinary least-squares regression fit, and the shaded area indicates the two-sided 95% confidence interval for the mean fitted relationship.

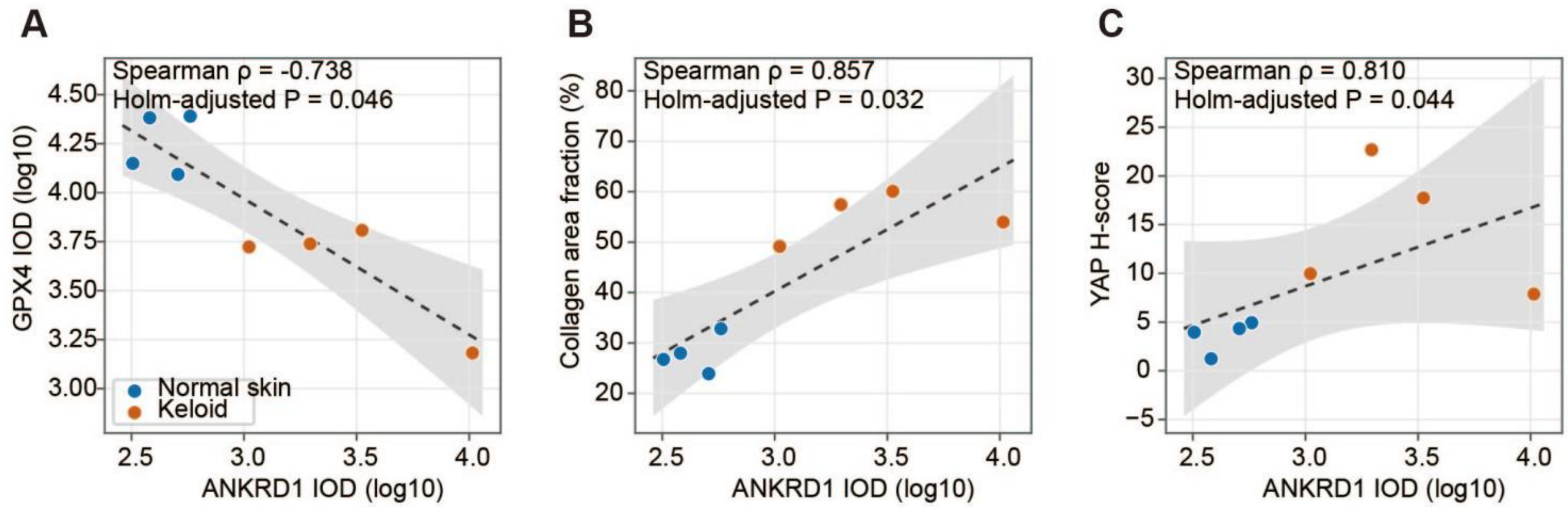

Supplementary Figure S4

Clinical tissue-level associations of ANKRD1 with GPX4, collagen burden and YAP. (A) Association between ANKRD1 and GPX4 immunofluorescence intensity. **(B)** Association between ANKRD1 immunofluorescence intensity and Masson-derived collagen area fraction. **(C)** Association between ANKRD1 immunofluorescence intensity and YAP H-score. Each point represents one tissue specimen, including four keloid and four normal-skin specimens. Colours indicate diagnostic group. IOD values are displayed on a log10 scale for visualization; correlation testing used the original specimen-level values. Spearman correlation coefficients and exact two-sided permutation P values were calculated, with Holm adjustment across the three displayed ANKRD1-centred comparisons. Dashed lines indicate ordinary least-squares fits and shaded areas indicate 95% confidence intervals.
